# Targeting CDK4/6 suppresses colorectal cancer by destabilizing YAP1

**DOI:** 10.1002/mco2.70103

**Published:** 2025-02-17

**Authors:** Yalei Wen, Xiao Yang, Shengrong Li, Lei Huang, Jiayi Chen, Lirong Tan, Xiuqing Ma, Yingjie Zhu, Zhengqiu Li, Changliang Shan, Chunze Zhang, Qiushi Zhang, Mingchao Liang, Haoxing Zhang, Tongzheng Liu

**Affiliations:** ^1^ Research Institute for Maternal and Child Health, The Affiliated Guangdong Second Provincial General Hospital, Postdoctoral Research Station of Traditional Chinese Medicine, School of Pharmacy Jinan University Guangzhou China; ^2^ State Key Laboratory of Bioactive Molecules and Druggability Assessment/International Cooperative Laboratory of Traditional Chinese Medicine Modernization and Innovative Drug Development of Ministry of Education (MOE) of China/College of Pharmacy Jinan University Guangzhou China; ^3^ State Key Laboratory of Medicinal Chemical Biology, College of Pharmacy and Tianjin Key Laboratory of Molecular Drug Research Nankai University Tianjin China; ^4^ Department of Colorectal Surgery, Tianjin Union Medical Center Nankai University Tianjin China; ^5^ Research Institute for Maternal and Child Health, The Affiliated Guangdong Second Provincial General Hospital Jinan University Guangzhou China; ^6^ The Affiliated Shunde Hospital of Jinan University Foshan China; ^7^ Guangdong Provincial Key Laboratory of Genome Stability and Disease Prevention, College of Life Sciences and Oceanography Shenzhen University Shenzhen China; ^8^ The State Key Laboratory of Functions and Applications of Medicinal Plants Guizhou Medical University Guiyang China

**Keywords:** abemaciclib, colorectal cancer (CRC), cyclin‐dependent kinase 4/6 (CDK4/6), deubiquitinating enzyme 3 (DUB3), Yes‐associated protein 1 (YAP1)

## Abstract

Colorectal cancer (CRC) is among the most prevalent and deadly cancers worldwide. The Yes‐associated protein 1 (YAP1) is frequently dysregulated in cancers, contributing to cancer stemness, chemoresistance, and cancer‐related death. However, strategies directly targeting YAP1 have not yet been successful because of the lack of active binding pockets and unregulated toxicity. In this study, our Food and Drug Administration (FDA)‐approved drug screening reveals that abemaciclib, a cyclin‐dependent kinase 4/6 (CDK4/6) inhibitor, dramatically promotes the proteasome‐dependent degradation of YAP1, thereby inhibiting tumor progression in CRC cells and patient‐derived xenograft models. We further identify deubiquitinating enzyme 3 (DUB3) as the bona fide deubiquitinase of YAP1 in CRC. Mechanistically, CDK4/6 directly phosphorylates DUB3 at Ser41, activating DUB3 to deubiquitinate and stabilize YAP1. Conversely, loss of Ser41 phosphorylation by CDK4/6 inhibition or Ser41A mutation, promotes YAP1 degradation and suppresses YAP1‐driven tumor progression. Histological analysis shows a positive correlation between DUB3 and YAP1 expression in CRC specimens. Collectively, our study uncovers a novel oncogenic role of the CDK4/6‐DUB3 pathway, which promotes YAP1 stabilization and tumor‐promoting function, highlighting that targeting CDK4/6 offers a potential therapeutic strategy for CRC with aberrantly upregulated DUB3 and YAP1.

## Graphical abstract

Our findings reveal a pivotal role of the cyclin‐dependent kinase 4/6–deubiquitinating enzyme 3 (CDK4/6–DUB3) axis in promoting the stabilization and tumor‐promoting function of Yes‐associated protein 1 (YAP1) and provide the preclinical evidence that CDK4/6 inhibitors might be an appealing therapeutic strategy for the treatment of colorectal cancer with aberrantly upregulated DUB3 and YAP1.

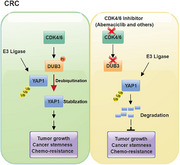

## 1 INTRODUCTION

Colorectal cancer (CRC) is among the most prevalent and deadly cancers worldwide.^1^ Despite advances in targeted and immune therapies, fluoropyrimidine‐based chemotherapeutic intervention remains one of the standard treatments for CRC, particularly in patients with advanced and metastatic diseases. However, frequent chemotherapy resistance is a significant cause of treatment failure and contributes to the high mortality rates in CRC patients.^2^, ^3^ Therefore, elucidating key mechanisms contributing to chemoresistance is crucial for discovering new therapeutic targets, which could greatly improve clinical outcomes for CRC patients.

Yes‐associated protein 1 (YAP1), a central effector of the Hippo pathway, plays a crucial role in regulating organ size, as well as controlling self‐renewal and differentiation of stem cells.^4^, ^5^, ^6^ Once YAP1 is phosphorylated by mammalian Sterile 20‐like kinase 1/2 (MST1/2) and large tumor suppressor 1 and 2 (LATS1/2), it is retained in the cytoplasm and targeted to degradation.^7^ Due to inactivation of Hippo signaling mediators, YAP1 is aberrantly stabilized or activated, which interacts with *TEADs*, *STAT3*, and other transcription factors, thereby inducing target genes expressions, and contributing to cancer stem‐like properties, tumorigenesis, and tumor progression.^8^, ^9^, ^10^, ^11^, ^12^ Given YAP1's unequivocal tumor‐promoting function in human cancer, it has gained recognition as an appealing therapeutic target. However, the development of YAP1 inhibitors has been limited.^13^ Some molecules, such as Verteporfin, CA3, TED‐347, and VGLL4‐mimicking peptides, disrupt TEAD–YAP1 interactions,^13^, ^14^, ^15^ while their clinical effectiveness are hindered by low plasma half‐life, poor cell‐penetrating ability, and limited specificity.^13^ The use of YAP1 antisense RNA has been evaluated for advanced solid tumors, however, the delivery of antisense RNA poses great challenges.^16^ Some small‐molecule inhibitors, such as dasatinib, along with statins and topoisomerase inhibitor A35, acted as effective YAP1 inhibitors in renal cell carcinoma and pancreatic cancer,^17^ while their effects on YAP1 were found to be nonspecific and indirect.^15^

Post‐translational modifications of YAP1, including phosphorylation and ubiquitination, have been shown to regulate its subcellular localization and degradation. E3 ubiquitin ligases, such as the F‐box and WD40 repeat domain containing‐7 (Fbxw7) and β‐TrCP destabilize YAP1 by targeting it for ubiquitination and proteasomal degradation in hepatocellular carcinoma (HCC) and pancreatic cancer.^18^, ^19^, ^20^ Conversely, deubiquitinase USP10 and USP9X stabilize YAP and promote cell growth and survival in HCC and breast cancer.^11^, ^21^ However, no direct modulators of E3s and DUBs of YAP1 have been clinically applied. Thus, identifying more actionable therapeutic drugs that destabilize YAP1 may significantly improve clinical outcomes in cancers with upregulated YAP1, including CRC.

Here, we identified abemaciclib, a cyclin‐dependent kinase 4/6 (CDK4/6) inhibitor, which led to more than an 80% decrease in YAP1 protein levels and suppressed CRC progression. Abemaciclib is a potent antiproliferative agent against HR^+^/HER2^−^ breast cancer cells by selectively inhibiting CDK4/6‐mediated phosphorylation of retinoblastoma protein (RB), halting the cell cycle progression from G1 phase to S phase, and suppressing tumor cell proliferation.^22^, ^23^ Moreover, CDK4/6 plays a critical role beyond its canonical function in cell cycle regulation, influencing various biological processes, including apoptosis, tumor growth, metastasis, and immunogenicity. These effects are mediated through the phosphorylation of various substrates by CDK4/6, including forkhead box m1 (FoxM1), feline mcdonough sarcoma (FMS)‐like tyrosine kinase 3 (FLT3), proviral integration site for Moloney murine leukemia virus 1 (PIM1) and tuberous sclerosis complex (TSC).^24^, ^25^, ^26^, ^27^ Here, we demonstrate a novel tumor‐promoting function of CDK4/6 in CRC by phosphorylating and activating deubiquitinating enzyme 3 (DUB3)‐mediated deubiquitination and stabilization of YAP1, suggesting that targeting CDK4/6 might be promising for managing CRC and other malignant cancers with dysregulated YAP1 and DUB3.

## 2 RESULTS

### 2.1 CDK4/6 inhibition induces YAP1 degradation in colorectal cancer

**FIGURE 1:**
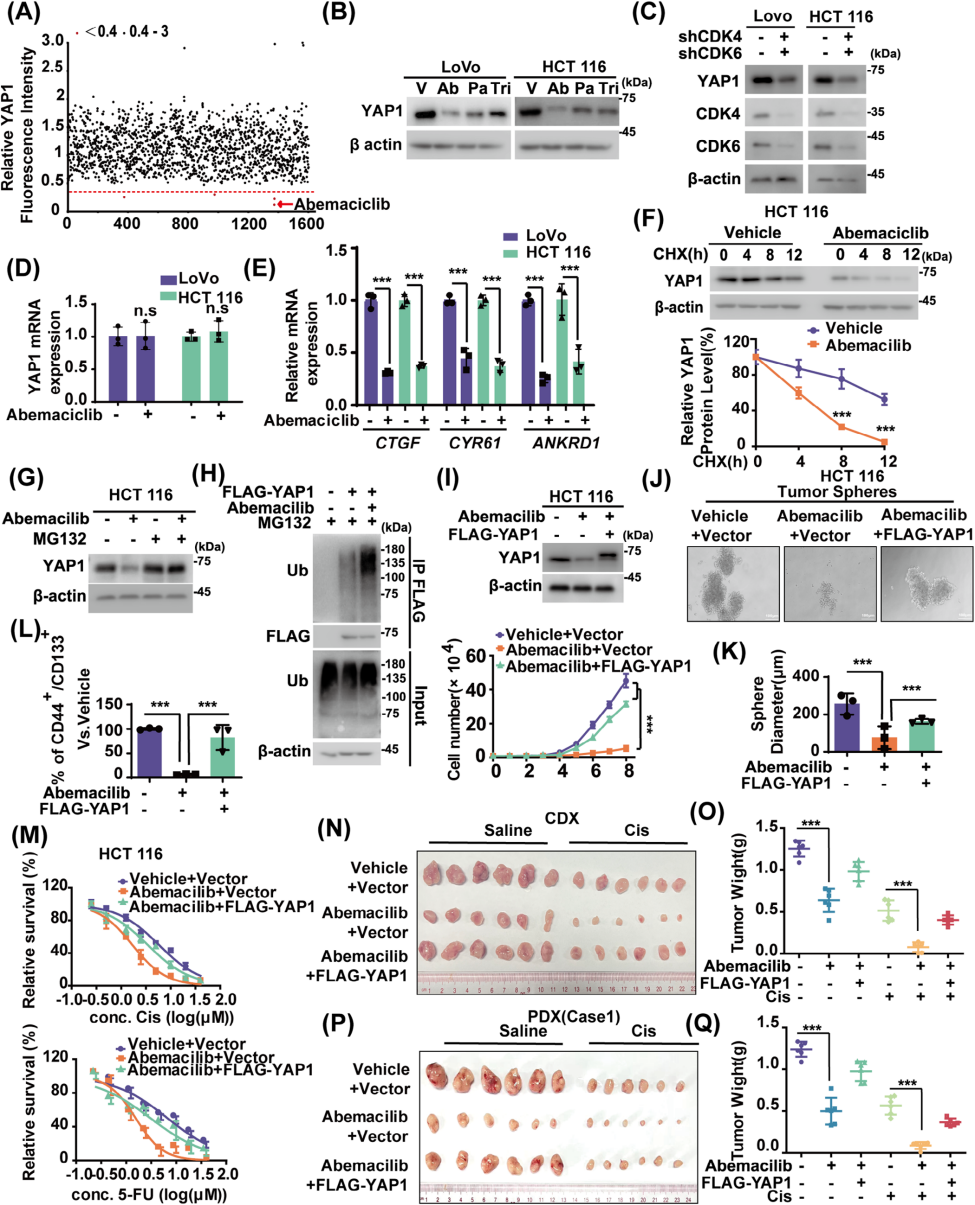
Cyclin‐dependent kinase 4/6 (CDK4/6) inhibition induces Yes‐associated protein 1 (YAP1) degradation and suppresses colorectal cancer (CRC) progression. (A) The high‐throughput screen of Food and Drug Administration (FDA)‐approved drug library identifies CDK4/6 inhibitor abemaciclib as a novel regulator of YAP1. (B) CRC cells were treated with vehicle or abemaciclib (Ab), palbociclib (Pa), and trilaciclib (Tri) at 5 µM for 24 h and western blotting was performed. (C) YAP1 in CRC cells stably expressing control (Ctrl) or shCDK4/6 was immunoblotted. (D) Cells were treated with vehicle or abemaciclib (5 µM) for 24 h and *YAP1* mRNA level was determined by quantitative real‐time PCR (qRT‐PCR). (E) CRC cells were treated with vehicle or abemaciclib (5 µM) and the transcription of YAP1 target genes was detected by qRT‐PCR. (F) Cycloheximide pulse‐chase assay was performed in the presence or absence of abemaciclib. The relative level of YAP1 to β‐actin was measured by image J. Results represent the mean ± SD of three independent experiments. (G) Cells were pretreated with vehicle or abemaciclib (5 µM) for 24 h and then treated with vehicle or MG132 (10 µM) for 10 h. YAP1 was immunoblotted. (H) Cells were transfected with indicated plasmids and treated with vehicle or abemaciclib (5 µM) for indicated period. Cell lysates were immunoprecipitated with anti‐FLAG affinity gel and immunoblotted. (I) Cells were transfected with indicated plasmids and treated with vehicle or abemaciclib (5 µM). Cell proliferation assay was performed. Results represent the mean ± SD of three independent experiments. (J, K) Tumor sphere formation abilities of HCT 116 cells as in (I) were measured and quantified (K). Scale bars, 100 µM. Results represent the mean ± SD of three independent experiments. (L) Graphic representation of the CD44^+^/CD133^+^ population from cells described in (I) was examined by fluorescence‐Activated Cell Sorting (FACS) analysis. Results represent the mean ± SD of three independent experiments. (M) Cells as in (I) were treated with indicated concentrations of cisplatin or 5‐fluorouracil (5‐FU) and cell survival was determined. The results represent mean ± SD from three independent experiments. (N, O) Cells as in (I) were subcutaneously implanted into nude mice (5–6 weeks, *n* = 6). When tumors reached around 150–200 mm^3^, mice were treated with saline, abemaciclib (50 mg/kg), cisplatin (2 mg/kg), or abemaciclib plus cisplatin. Tumors were collected (N) and tumor weights were analyzed (O). The results represent the mean ± SD of data from six mice. (P, Q) CRC patient‐derived xenografts (PDXs) were subcutaneously implanted into nude mice (5–6 weeks, *n* = 6). When tumors reached around 150–200 mm^3^ in size, mice were treated with saline, abemaciclib (50 mg/kg), cisplatin (2 mg/kg), or abemaciclib plus cisplatin (*n* = 6). Tumors were collected and tumor weights were analyzed. ^ns^ *p* > 0.05; ^*^p < 0.05; ^**^p < 0.01; ^***^p < 0.001 by Student's *t*‐test or one‐way analysis of variance (ANOVA) with Tukey's post hoc test.

YAP1 expressions in CRC tissues were measured using immunohistochemistry (IHC). As shown in Figure S1A, YAP1 protein levels in CRC specimens were higher than in paired adjacent normal tissues. YAP1 depletion significantly reduced cell proliferation and increased cellular sensitivities to cisplatin and 5‐fluorouracil (5‐FU), common chemotherapeutic drugs for CRC (Figure S1B,C). Although Verteporfin could inhibit the association of YAP1‐TEADs and suppress liver overgrowth and tumorigenesis driven by YAP1 overexpression or neurofibromatosis 2 (NF2)/Merlin inactivation,^28^ challenges such as identifying responsive patient populations and addressing toxicity issues have limited its clinical implementation as a YAP1 inhibitor.^29^ Thus, we screened Food and Drug Administration (FDA)‐approved drugs to mitigate YAP1 stability by assessing fluorescence intensity in endogenous YAP1‐deficient HCT 116 cells stably expressing green fluorescent protein (GFP)‐YAP1. As shown in Figure 1A, abemaciclib, a drug approved for treating HR^+^/HER2^−^ advanced breast cancer through CDK4/6 inhibition,^30^ significantly reduced the fluorescence intensity of GFP‐YAP1. Additionally, CDK4/6 inhibitors palbociclib and trilaciclib reduced YAP1 protein level in HCT 116 and LoVo cells with relatively weaker inhibitory effects (Figures 1B and S1D). Consistently, simultaneous depletion of CDK4 and CDK6 in LoVo and HCT 116 cells significantly decreased YAP1 protein level and mRNA levels of YAP1 target genes connective tissue growth factor (*CTGF*), cysteine‐rich angiogenic inducer 61 (*CYR61*), and Ankyrin repeat domain 1 (*ANKRD1*), while transcript levels of YAP1 itself were not affected (Figure 1C–E). Intriguingly, cycloheximide pulse‐chase （CHX） assay showed that YAP1 protein was less stable in HCT 116 cells treated with abemaciclib (Figure 1F). Additionally, MG132, a proteasome inhibitor, could restore decreased YAP1 levels caused by CDK4/6 inhibition (Figure 1G), which might be attributed to elevated YAP1 ubiquitination levels (Figures 1H and S1E). These findings suggest that targeting CDK4/6 destabilizes YAP1 by a proteasome‐dependent mechanism.

We next investigated whether targeting CDK4/6 in CRC could regulate YAP1‐dependent malignancies. As shown in Figures 1I and S1F, CDK4/6 inhibition by abemaciclib significantly suppressed cell proliferation in LoVo and HCT 116 cells. Growing evidence indicates that cancer stem cells (CSCs) play a crucial role in developing chemoresistance.^31^ Given that YAP1 has been showed to enhance CSC characteristics,^32^ the effect of CDK4/6 on the self‐renewal capacity of CSCs were examined in CRC. As shown in Figure 1J–L, abemaciclib treatment in HCT 116 cells significantly suppressed mammosphere formation and decreased the proportion of CD44^+^/CD133^+^ cells, which are recognized as markers of CRC CSC population.^33^, ^34^ The primary mechanism for targeting CDK4/6 in HR^+^/HER2^−^ advanced breast cancer involves inhibiting RB phosphorylation, resulting in cell cycle arrest.^35^, ^36^ We also investigated whether targeting CDK4/6 could regulate the cell cycle in CRC. As shown in Figure S1G, abemaciclib treatment in LoVo cells significantly induced G1 cell cycle arrest. Additionally, CDK4/6 inhibition or depletion in LoVo and HCT 116 cells significantly inhibited cell growth and enhanced cellular sensitivity to cisplatin and 5‐FU (Figures 1M and S1H–K). Meanwhile, reconstituting YAP1 markedly restored the phenotypic changes induced by CDK4/6 inhibition or depletion (Figures 1I–M and S1F–K). We observed comparable outcomes in both CRC cancer cell line xenograft (CDX) and two patient‐derived tumor xenograft (PDX) models (Figures 1N–Q and S1L). We also found that CDK4/6 inhibition by abemaciclib dramatically reduced YAP1 expressions while concurrently upregulating cleaved‐poly(ADP‐ribose) polymerase‐1 (PARP1) levels in tumor samples from saline‐treated mice. These effects were even more pronounced in the cisplatin‐treated group (Figure S1M). Consistently to in vitro results, reconstituting YAP1 markedly rescued these effects of CDK4/6 inhibition.

CDK4 and CDK6 are closed related kinases, sharing about 71% amino acid homology. Both kinases associate with cyclin D1‐3, performing many overlapping functions by phosphorylating RB, TSC2, and other protein substrates.^37^ We further examined the relative effect of knocking down CDK4 or CDK6 alone on YAP1 protein level in CRC. As shown in Figure S1N, depletion of either CDK4 or CDK6 in HCT 116 cells led to a moderate decrease in YAP1 protein levels, while simultaneous knockdown of both kinases resulted in a more significant reduction. Functionally, depletion of CDK4 or CDK6 partially inhibited cell proliferation, whereas simultaneous knockdown of both kinases displayed a more pronounced inhibitory effect.

YAP1 induces expressions of *CTGF*, *CYR61*, and other target genes, contributing to cancer stemness, chemoresistance, metastasis, and poor prognosis.^38^ CTGF increases matrix metalloproteinase expression, promoting tumor proliferation and chemoresistance.^39^ CYR61 plays diverse roles in promoting cellular proliferation, survival, and differentiation.^38^ We next investigated whether YAP1 target genes are involved in tumor‐promoting function of CDK4/6 in CRC. As shown in Figure S1O–R, CDK4/6 inhibition by abemaciclib in LoVo cells significantly decreased YAP1 protein levels and expressions of target genes, resulting in reduced cell proliferation and enhanced cellular responsiveness to cisplatin and 5‐FU. Interestingly, reconstituting either CTGF or CYR61 partially rescued these phenotypic changes induced by CDK4/6 inhibition. These results showed that CDK4/6 inhibition suppresses CRCs mainly through destabilizing YAP1 and subsequently impairing expressions of target genes.

### 2.2 Identification of the DUB3 as the bona fide deubiquitinase of YAP1

**FIGURE 2:**
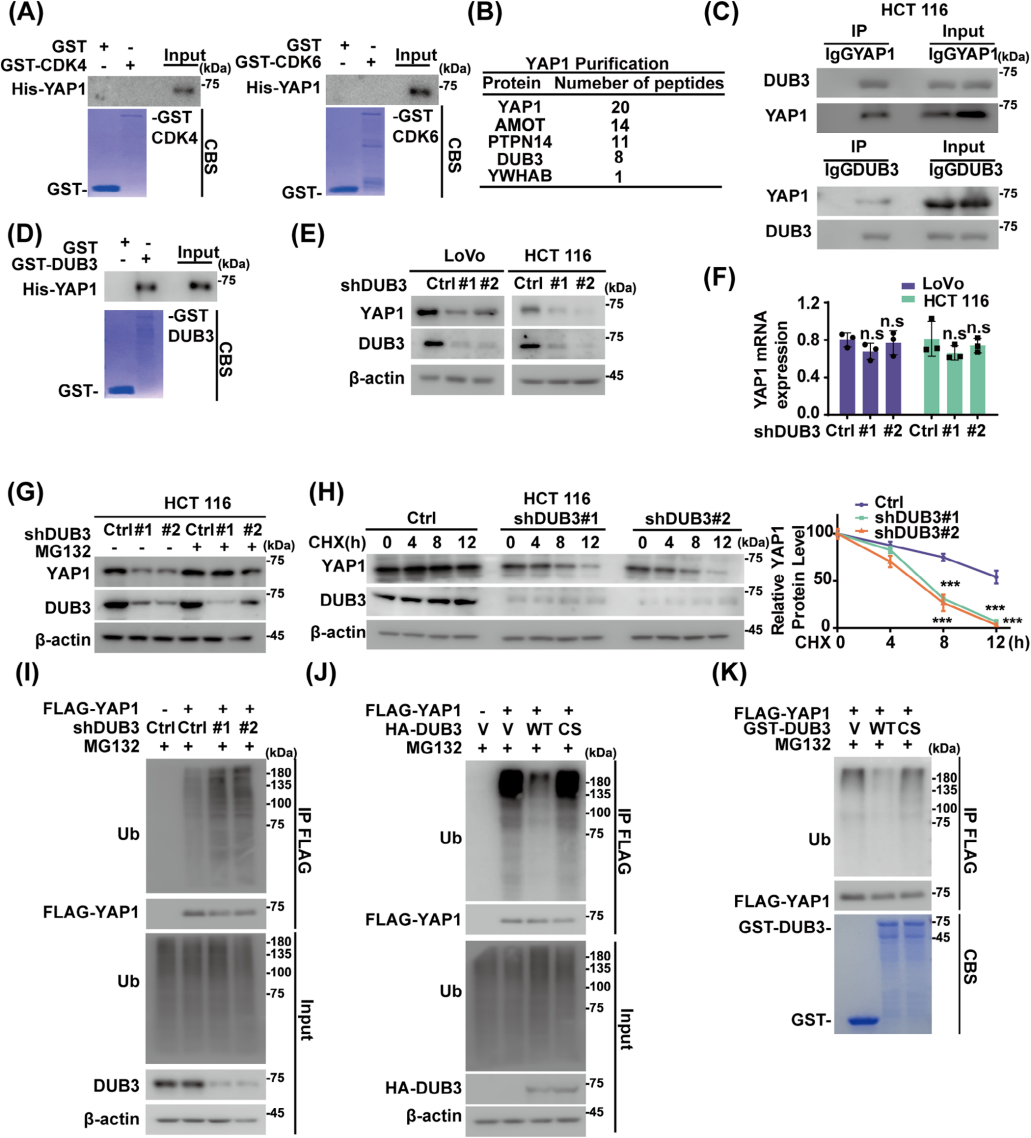
Deubiquitinating enzyme 3 (DUB3) deubiquitinates and stabilizes Yes‐associated protein 1 (YAP1). (A) Purified recombinant His‐YAP1 were incubated with GST or GST‐cyclin‐dependent kinase 4/6 (CDK4/6) and the direct interaction between YAP1 and CDK4/6 was examined. CBS, Coomassie blue staining. (B) List of YAP1‐associated proteins identified by mass spectrometric analysis. HCT 116 cells stably expressing FLAG‐YAP1 were generated and YAP1 immunoprecipitates were subjected to mass spectrometric analysis. (C) Cell lysates of HCT 116 were subjected to immunoprecipitation with IgG, anti‐YAP1, or anti‐DUB3 antibodies, respectively. Western blotting was performed with indicated antibodies. (D) Purified recombinant His‐YAP1 were incubated with GST or GST‐DUB3 and the direct interaction between YAP1 and DUB3 was examined. CBS, Coomassie blue staining. (E) LoVo and HCT 116 cells stably expressing control (Ctrl), shDUB3#1, shDUB3#2 were generated. Protein levels of DUB3 and YAP1 were measured by western blotting. (F) Total RNA was isolated from cells in (E). The expression of *YAP1* mRNA in cells was determined by quantitative real‐time PCR (qRT‐PCR). Transcript levels were determined as the relative expression level to the *GAPDH* mRNA and normalized as the percentage of the control respectively. The results were represented as mean ± SD from three independent experiments. (G) HCT 116 in (E) cells stably expressing control, shDUB3#1, shDUB3#2 were treated with vehicle or MG132 (10 µM) and western blotting was performed with indicated antibodies. (H) Cycloheximide pulse‐chase assay was performed in HCT 116 cells as in (E); the relative level of YAP1 to β‐actin was measured by image J. The results represent mean ± SD from three independent experiments. (I) HCT 116 cells stably expressing control, shDUB3#1, shDUB3#2 were transfected as indicated and treated with MG132 (10 µM) for 10 h. Cell lysates were immunoprecipitated with anti‐FLAG affinity gel and immunoblotted as indicated. (J) HCT 116 cells were cotransfected with vector, FLAG‐YAP1, HA‐DUB3 WT, or HA‐DUB3 C89S mutant as indicated, then were treated MG132 (10 µM) for 10 h. Cell lysates were immunoprecipitated with anti‐FLAG affinity gel and immunoblotted as indicated. (K) HCT 116 cells were transfected with FLAG‐YAP1 and treated with MG132 (10 µM) for 10 h. YAP1 was immunoprecipitated with anti‐FLAG affinity gel and incubated with purified GST, GST‐DUB3 WT, or GST‐DUB3 C89S. The polyubiquitylated YAP1 protein was detected. ^ns^ *p* > 0.05; ^*^p < 0.05; ^**^p < 0.01; ^***^p < 0.001 by one‐way analysis of variance (ANOVA) with Tukey's post hoc test.

CDK4/6 directly phosphorylates and regulates the degradation of some substrates such as FoxM1.^24^ However, we did not detect any interaction of purified GST‐CDK4/6 and His‐YAP1 (Figure 2A), suggesting that CDK4/6 might regulate YAP1 through a yet‐to‐be‐identified intermediary factor instead of acting directly on YAP1. Tandem affinity purification and mass spectrometry analysis were next performed using HCT 116 cells stably expressing FLAG‐S‐YAP1. Along with other previously identified YAP1‐binding proteins, including angiomotin (AMOT)^40^ and protein tyrosine phosphatase, nonreceptor type 14 (PTPN14),^41^ the DUB3 was identified as a potential interactor of YAP1 (Figure 2B). Endogenous interactions of DUB3–YAP1 were next validated in HCT 116 and LoVo cells through a coimmunoprecipitation assay (Figures 2C and S2A). Furthermore, purified GST‐DUB3, but not GST alone, was able to bind to YAP1 under cell‐free conditions (Figure 2D).

The role of DUB3 in regulating YAP1 was next investigated. As shown in Figure 2E,F, DUB3 depletion in HCT 116 and LoVo cells significantly decreased YAP1 protein levels without affecting its mRNA levels, meanwhile MG132 restored the reduced YAP1 protein levels caused by DUB3 depletion (Figures 2G and S2B). Consistent with these findings, YAP1 exhibited reduced stability in DUB3‐depleted cells (Figure 2H), likely due to increased polyubiquitination levels of YAP1 (Figure 2I). Furthermore, DUB3 WT significantly reduced polyubiquitination levels of YAP1, while the catalytically inactive mutant DUB3 C89S failed to do that (Figure 2J). Additionally, incubation with purified GST‐DUB3 WT in vitro, in contrast to the C89S mutant, caused a notable reduction in polyubiquitinated YAP1 (Figure 2K). The specific ubiquitin linkage on YAP1 cleaved by DUB3 were further examined. Both K48‐ and K63‐linked polyubiquitin chains were detected on YAP1, but DUB3 only selectively cleaved the K48‐linked polyubiquitin chains on YAP1 (Figure S2C). We then introduced point mutations into YAP1 at several putative ubiquitination sites on YAP1, as predicted by PhosphoSitePlus database. As shown in Figure S2D,E, single mutant K181R, K315R or K497R moderately decreased YAP1 ubiquitination, while the K181R/K315R/K497R mutant significantly increased YAP1 stability compared to YAP1 WT. These findings indicate that DUB3 specifically deubiquitinates YAP1 in CRC.

### 2.3 DUB3 promotes colorectal cancer progression through stabilizing YAP1

**FIGURE 3:**
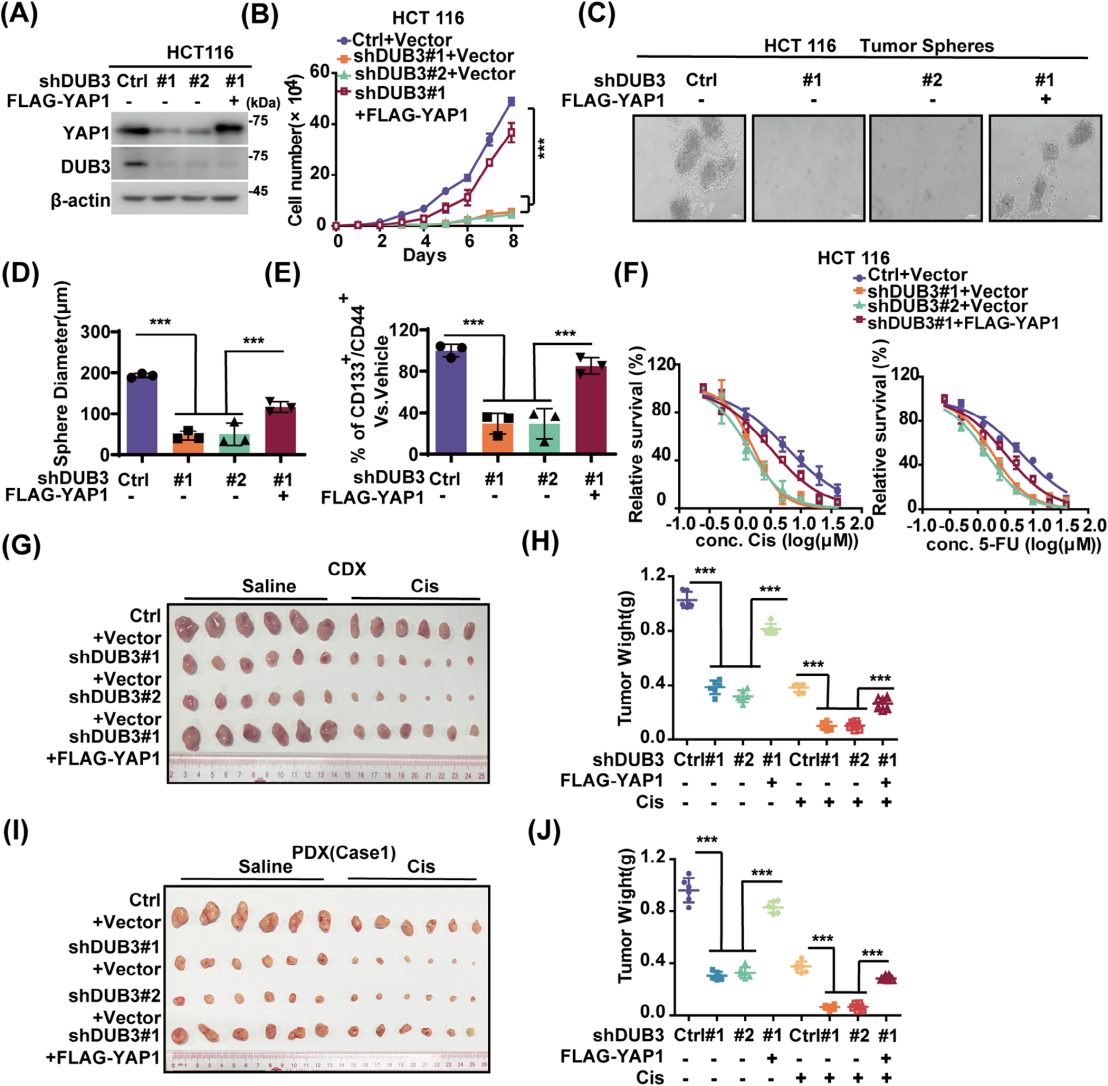
Deubiquitinating enzyme 3 (DUB3) promotes colorectal cancer progression through stabilizing Yes‐associated protein 1 (YAP1). (A) HCT 116 cells stably expressing Ctrl, shDUB3#1, shDUB3#2 were transfected with vector or FLAG‐YAP1and western blotting was performed with indicated antibodies. (B) Cell proliferation of HCT 116 cells in (A) was examined. Results represent the mean ± SD of three independent experiments. (C, D) Tumor sphere formation abilities in HCT 116 cells as in (A) were measured (C). Scale bars=100 µM. Results were represented as the mean ± SD of three independent experiments (D). (E) Graphic representation of the CD44^+^/CD133^+^ population of HCT 116 cells as in (A) was examined by FACS analysis. Results represent the mean ± SD of three independent experiments. (F) HCT 116 cells as in (A) were treated with indicated concentrations of cisplatin or 5‐fluorouracil (5‐FU) and cell survival was determined. Results represent mean ± SD from three independent experiments. (G, H) HCT 116 cells as in (A) were subcutaneously implanted into nude mice (5–6 weeks, *n* = 6). When tumors reached around 150–200 mm^3^ in size, mice were treated with saline or cisplatin (2 mg/kg). Tumors were collected (G) and weights were measured (H). The results represent the mean ± SD of data from six mice (5–6 weeks, *n* = 6). (I, J) Colorectal cancer patient‐derived xenografts (PDX case 1) were subcutaneously implanted into nude mice (5–6 weeks, *n* = 6). Xenograft tumors were injected with lentivirus expressing the indicated constructs when tumor volume reached 30–50 mm^3^. Mice were then treated with saline or cisplatin (2 mg/kg), respectively. Tumors were collected and tumor weights were analyzed. Xenograft tumors were dissected at indicated time and tumor weights were measured in right panel. Results represent the mean ± SD from six mice. ^ns^ *p* > 0.05; ^*^p < 0.05; ^**^p < 0.01; ^***^p < 0.001 by one‐way analysis of variance (ANOVA) with Tukey's post hoc test.

Considering the well‐established oncogenic role of YAP1^42^ and its stability by DUB3 (Figure 2), roles of DUB3 in CRC progression were explored. DUB3 depletion in LoVo and HCT 116 cells significantly decreased YAP1 protein levels, suppressed cell proliferation and sphere formation, as well as reduced stem cell proportion of CD44^+^/CD133^+^ cells (Figures 3A–F and S3A,B). Additionally, DUB3 depletion increased cellular sensitivity to cisplatin or 5‐FU. Notably, reconstituting YAP1 in DUB3‐deficient cells markedly rescued these effects. Consistent results were observed in HCT 116 cell‐based xenograft and two CRC PDX xenograft models (Figures 3G–J and S3C). As shown in Figure S3D, DUB3 depletion dramatically reduced YAP1 expressions while concurrently upregulated cleaved‐PARP1 levels in tumor samples from mice treated with saline. These effects were even more pronounced in the cisplatin‐treated group. Consistently to in vitro results, reconstituting YAP1 markedly rescued these effects caused by DUB3 depletion. We next investigated whether YAP1 target genes were involved in the role of the DUB3–YAP1 axis in CRC. DUB3 depletion in LoVo cells significantly decreased YAP1 protein levels and expressions of *CTGF* and *CYR61*, resulting in reduced cell growth and enhanced cellular responsiveness to cisplatin and 5‐FU (Figure S3E–H). Interestingly, reconstituting CTGF or CYR61 in DUB3‐depleted LoVo cells partially rescued the phenotypic changes caused by DUB3 depletion. Together, our results uncover a novel tumor‐promoting role of DUB3 in CRC mainly through stabilizing YAP1.

### 2.4 CDK4/6 phosphorylates DUB3 at Ser41

**FIGURE 4:**
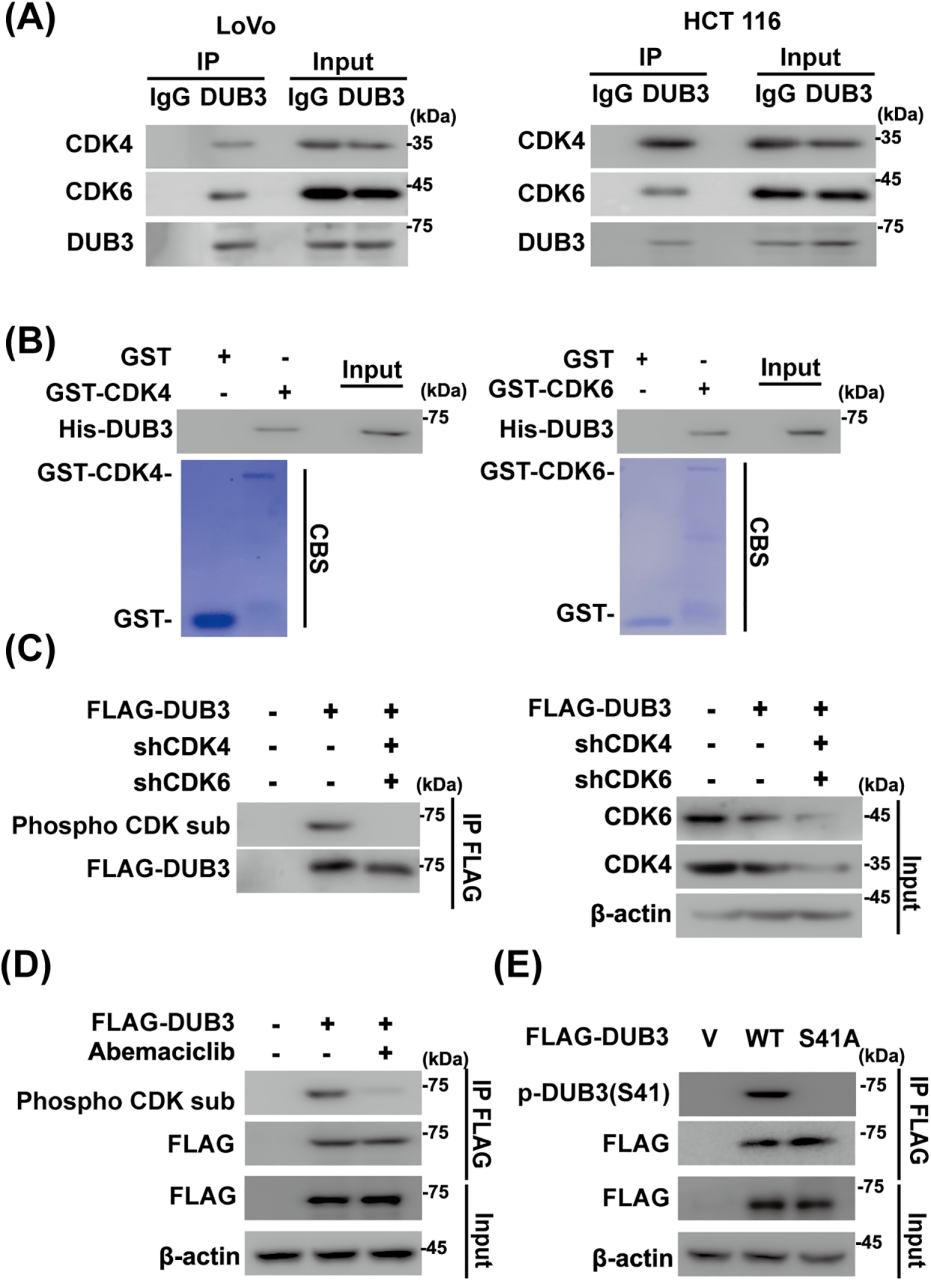
Cyclin‐dependent kinase 4/6 (CDK4/6) phosphorylates deubiquitinating enzyme 3 (DUB3) at Ser41. (A) LoVo and HCT 116 cell lysates were subjected to immunoprecipitation with IgG, anti‐DUB3 antibodies. The immunoprecipitates were blotted with indicated antibodies. (B) Purified recombinant GST, GST‐CDK4/6 and His‐DUB3 were incubated in vitro as indicated. The interaction between CDK4/6 and DUB3 was examined. CBS, Coomassie blue staining. (C) HCT 116 cells stably expressing Ctrl or shCDK4/6 were generated and transfected with vector or FLAG‐DUB3. Cell lysates were subjected to immunoprecipitation with anti‐FLAG affinity gel, and the phosphorylation of DUB3 were examined by using phospho‐CDK substrate antibody. (D) HCT 116 cells were transfected with vector or FLAG‐DUB3 and then treated with vehicle or abemaciclib (5 µM). Cell lysates were immunoprecipitated with anti‐FLAG affinity gel and the phosphorylation of DUB3 was examined by phospho‐CDK substrate antibody. (E) HCT 116 cells were transfected with vector, FLAG‐DUB3 WT or S41A. Cell lysates were immunoprecipitated with anti‐FLAG affinity gel and the phosphorylation of DUB3 was then examined with anti‐phospho‐Ser41 antibody.

Based on the aforementioned results, we hypothesized that DUB3 might serve as a potential connector between CDK4/6 and YAP1 in CRC. The CDK4/6‐DUB3 interaction were first validated in CRC cells and under cell‐free conditions (Figure 4A,B). Next, we investigated phosphorylation of DUB3 by CDK4/6 in CRC. DUB3 phosphorylation was detected in HCT 116 cells using phospho‐CDK substrate antibody, which was significantly reduced upon CDK4/6 depletion and inhibition (Figure 4C,D). Our previous study demonstrated that DUB3 was phosphorylated at Ser41 by CDK4/6, promoting cancer metastasis of triple‐negative breast cancer (TNBC).^43^ We then tested whether CDK4/6 could catalyze the phosphorylation of DUB3 at the same site in CRC cells. As shown in Figure 4E, DUB3 WT rather than the S41A mutant could be phosphorylated in HCT 116 cells using the phospho‐Ser41‐specific antibody, indicating that CDK4/6 directly phosphorylate DUB3 in CRC.

### 2.5 Phosphorylation of DUB3 by CDK4/6 is pivotal for YAP1 stability and colorectal cancer progression

**FIGURE 5:**
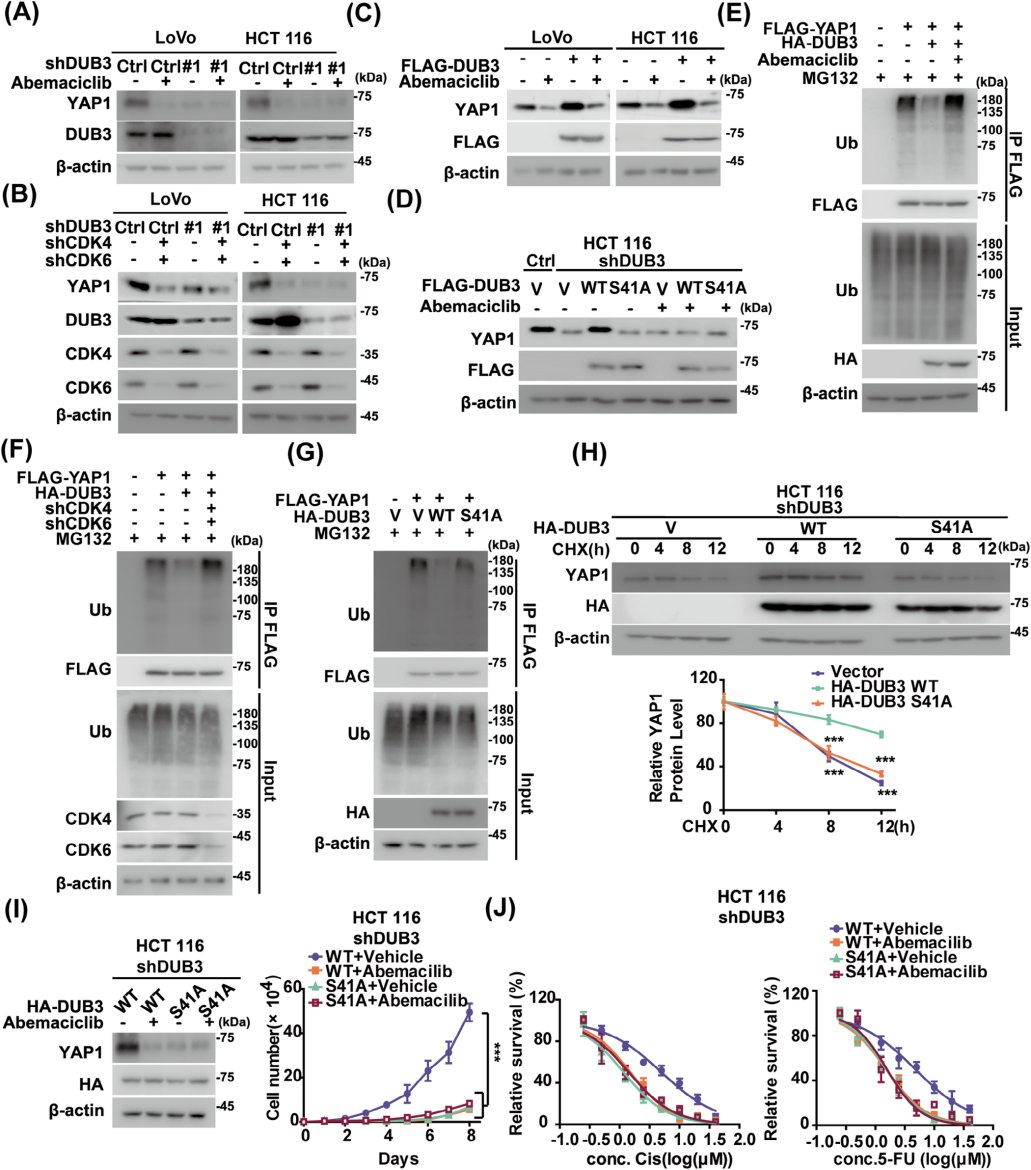
Cyclin‐dependent kinase 4/6 (CDK4/6)‐mediated phosphorylation of deubiquitinating enzyme 3 (DUB3) is pivotal for Yes‐associated protein 1 (YAP1) stability and colorectal cancer progression. (A) LoVo and HCT 116 cells stably expressing control or shDUB3#1 were treated with vehicle or abemaciclib (5 µM). Western blotting was performed. (B) LoVo and HCT 116 cells stably expressing control, shDUB3#1, or shCDK4/6 were generated and western blotting was performed. (C) LoVo and HCT 116 cells stably expressing vector or FLAG‐DUB3 were treated with vehicle or abemaciclib (5 µM) and YAP1 protein levels were examined. (D) HCT 116 cells stably expressing control or shDUB3#1 were transfected with vector, DUB3 WT, or the phosphorylation‐defective mutant S41A. Cells were treated with vehicle or abemaciclib (5 µM) and western blotting was performed. (E) HCT 116 cells were transfected with indicated plasmids and treated with vehicle or abemaciclib (5 µM). Cells were then treated MG132 (10 µM) for 10 h. Cell lysates were immunoprecipitated with anti‐FLAG affinity gel and immunoblotted as indicated. (F) Cells were transfected with indicated plasmids and then treated with MG132 (10 µM) for 10 h. Cell lysates were immunoprecipitated with anti‐FLAG affinity gel and immunoblotted as indicated. (G) Cells were cotransfected with FLAG‐YAP1 and other indicated plasmids. Cells were treated MG132 (10 µM) for 10 h. Cell lysates were immunoprecipitated with anti‐FLAG affinity gel and immunoblotted as indicated. (H) Endogenous DUB3‐deficient HCT 116 cells were transfected with vector, DUB3 WT, or the S41A mutant and cycloheximide pulse‐chase assay was performed. The relative level of YAP1 to β‐actin was measured by image J. The results represent mean ± SD from three independent experiments. (I) Endogenous DUB3‐deficient HCT 116 cells were transfected with indicated plasmids. The protein level of YAP1 was determined by western blot and cell proliferation was measured. The results represent mean ± SD from three independent experiments. (J) HCT 116 cells as in (I) were treated with indicated concentrations of cisplatin or 5‐fluorouracil (5‐FU) and cell survival was determined. The results represent mean ± SD from three independent experiments. ^ns^ *p* > 0.05; ^*^p < 0.05; ^**^p < 0.01; ^***^p < 0.001 by one‐way analysis of variance (ANOVA) with Tukey's post hoc test.

We next explored whether DUB3 is the main mediator of YAP1 regulation by CDK4/6 in CRC. As shown in Figure 5A,B, DUB3 depletion and CDK 4/6 inhibition by abemaciclib both significantly reduced YAP1 protein levels, while the combination did not result in any further reduction of YAP1 levels. Additionally, overexpression of DUB3 WT in LoVo and HCT 116 cells significantly increased YAP1 protein levels, which was largely mitigated by the pretreatment of abemaciclib (Figure 5C).

We then examined whether CDK4/6‐mediated phosphorylation influence DUB3's ability to stabilize YAP1. Compared to the S41A mutant, reintroducing DUB3 WT into endogenous DUB3‐deficient cells resulted in increased YAP1 protein levels (Figure 5D). Interestingly, abemaciclib dramatically decreased YAP1 protein levels in cells reconstituting DUB3 WT, but had no effect in those with S41A. The effect of DUB3 phosphorylation by CDK4/6 on YAP1 ubiquitination were next assessed. DUB3 WT markedly reduced YAP1 ubiquitination levels, whereas CDK4/6 depletion or inhibition blocked it (Figures 5E,F and S4A). In contrast, the S41A mutant hardly influence YAP1 ubiquitination levels regardless of CDK4/6 inhibition (Figure 5G). Moreover, reintroducing DUB3 WT into HCT 116 cells depleting endogenous DUB3 significantly stabilized YAP1 compared with S41A (Figure 5H). This suggests that DUB3 phosphorylation catalyzed by CDK4/6 is essential for YAP1 stability in CRC.

We then explored the effects of DUB3 phosphorylation by CDK4/6 on YAP1‐driven oncogenic processes. Reconstituting DUB3 WT in endogenous DUB3‐deficient HCT 116 and LoVo cells significantly enhanced cell proliferation and decreased sensitivity to cisplatin and 5‐FU, compared to the DUB3 S41A mutant (Figures 5I,J and S4B,C). These effects were significantly inhibited by CDK4/6 inhibitor abemaciclib. Conversely, reconstituting the DUB3 S41A mutant exhibited a strong inhibitory effect on these malignant processes, which was not affected by CDK4/6 inhibition. Altogether, these findings demonstrate that DUB3 phosphorylation by CDK4/6 is critical for YAP1‐driven CRC progression.

### 2.6 Aberrantly upregulated DUB3 positively correlates with YAP1 expression in colorectal cancer

**FIGURE 6:**
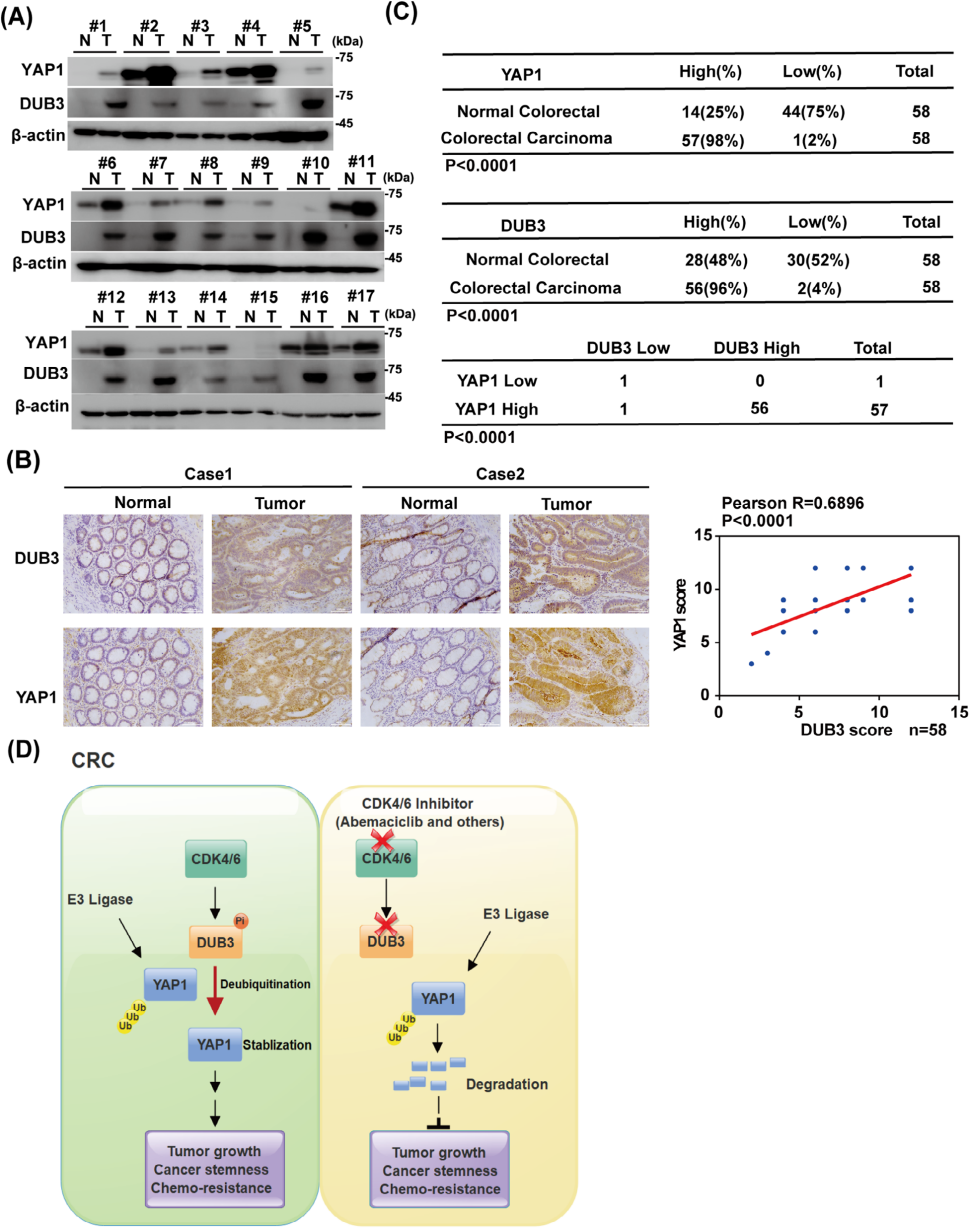
Aberrantly upregulated deubiquitinating enzyme 3 (DUB3) positively correlates with Yes‐associated protein 1 (YAP1) expression in colorectal cancer (CRC). (A) Western blot analysis of YAP1 and DUB3 expression in 17 paired CRC specimens and adjacent normal tissues. N, adjacent normal tissue; T, tumor tissue. (B) Representative images of immunohistochemical staining of DUB3 and YAP1 in 58 cases of CRC and adjacent normal tissues. Correlation of YAP1 expression with DUB3 was analyzed in right panel. Statistical analyses were performed with the Fisher's exact test. (C) the correlation of DUB3 and YAP1 expressions in CRC tissues were analyzed by the *χ* ^2^‐test. Statistical analyses. *R*, the Pearson correlation coefficient. (D) Working model to illustrate that cyclin‐dependent kinase 4/6 (CDK4/6)‐mediated activation of DUB3 in CRC regulates tumor proliferation, self‐renewal, and chemoresistance through stabilizing YAP1.

To evaluate the clinical significance of this pathway in CRC, we performed immunoblotting and IHC staining. Both DUB3 and YAP1 expressions in CRC specimens were markedly higher than in the adjacent normal tissues (Figure 6A,B). Furthermore, higher DUB3 expressions were positively associated with higher YAP1 expressions (Figure 6C). Additionally, the relationship between YAP1 and DUB3 expression and the clinical features of CRC patients were evaluated. Receiver operating characteristic (ROC) curve analysis revealed that the optimal cut‐off score of YAP1 and DUB3 expression to predict advanced stage, with high sensitivity and specificity, was 10.5 (Figure S5A,B). Based on the cut‐off value, patients were subsequently categorized into high‐expression and low‐expression groups with DUB3 and YAP1. As shown in Table 1, higher DUB3 expression was positively associated with higher TN stages, and more advanced clinical stages (*p* <  0.05), but not correlated with age, gender, tumor size, and location. Similarly, higher YAP1 expression was associated with TN stage, advanced clinical stage, and poorer tumor differentiation (*p* <  0.05), but showed no correlation with age, gender, tumor size, or location. Collectively, our preclinical study suggests that targeting CDK4/6 could serve as a promising therapeutic approach for treating CRC and other cancers characterized by overexpression of wild‐type DUB3 and YAP1 (Figure 6D).

**TABLE 1:** Relationship between Yes‐associated protein 1 (YAP1) and deubiquitinating enzyme 3 expression and the clinical characteristics of colorectal cancer patients.

		DUB3 expression				YAP1 expression			
Clinical character	Number	Low (<10.5)	High (≥10.5)	*χ* ^2^	*p* value	Low (<10.5)	High (≥10.5)	*χ* ^2^	*p* value
Gender									
Gender	31	19 (61.3%)	12 (38.7%)	0.017	0.896	18 (58.1%)	13 (41.9%)	0.037	0.847
Male	27	17 (63.0%)	10 (37.0%)			15 (55.6%)	12 (44.4%)
Age (year)									
<60	28	18 (64.3%)	10 (35.7%)	0.113	0.737	17 (60.7%)	11 (39.3%)	0.322	0.571
≥60	30	18 (60.0%)	12 (40.0%)			16 (53.3%)	14 (46.7%)
Size									
<5 cm	49	28 (57.1%)	21 (42.9%)	2.046	0.153	30 (61.2%)	19 (38.8%)	1.409	0.235
≥5 cm	9	8 (88.9%)	1 (11.1%)			3 (33.3%)	6 (66.7%)
Location									
Right hemicolon	8	7 (87.5%)	1 (12.5%)	1.450	0.228	5 (62.5%)	3 (37.5%)	<0.001	>0.999
Left hemicolon	50	29 (58.0%)	21 (42.0%)			28 (56.0%)	22 (44.0%)
Differentiated degree									
High differentiation	19	16 (84.2%)	3 (15.8%)	4.568	0.033	17 (89.5%)	2 (10.5%)	10.332	<0.001
Mid–low differentiation	39	20 (51.3%)	19 (48.7%)			16 (41.0%)	23 (59.0%)
T (tumor) stage									
Tis–T2	32	27 (84.4%)	5 (15.6%)	15.086	<0.001	28 (87.5%)	4 (12.5%)	24.549	<0.001
T3–T4	26	9 (34.6%)	17 (65.4%)			5 (19.2%)	21 (80.8%)
N (node) stage									
N0	32	27 (84.4%)	5 (15.6%)	15.086	<0.001	28 (87.5%)	4 (12.5%)	24.549	<0.001
N1–N2	26	9 (34.6%)	17 (65.4%)			5 (19.2%)	21 (80.8%)
Clinical stage									
I–II	32	27 (84.4%)	5 (15.6%)	15.086	<0.001	28 (87.5%)	4 (12.5%)	24.549	<0.001
III–IV	26	9 (34.6%)	17 (65.4%)			5 (19.2%)	21 (80.8%)

## 3 DISCUSSION

The role of YAP1 in maintaining normal cellular functions is under precise and tight regulation. However, frequent dysregulations of YAP1 contributes to cancer stemness, chemoresistance and increased cancer‐related mortality, making YAP1 a promising drug target.^44^ However, targeting YAP1 remains challenging and strategies aimed directly at YAP1 have not yet been successful in clinical management. One strategy for targeting YAP1 activity is to inhibit its interaction with transcriptional factors TEADs. Verteporfin, an FDA‐approved drug, can effectively disrupt this interaction. However, its clinical potential for treating YAP1‐TEAD‐driven cancers is constrained by off‐target effects. Cyclic YAP‐like peptides, IAG933, palmitoylation inhibitors, and other allosteric inhibitors also disrupt the YAP–TEAD interaction,^29^ however, no clinical data are yet available regarding their potential efficacy in cancer treatment. Strategies targeting YAP1 downstream effectors such as protein kinases, metabolic enzymes, ligands and proteins like B‐cell lymphoma‐extra‐large (Bcl‐xL), FoxM1, and transglutaminase (TG2) are also being explored to counteract YAP1‐mediated malignancies. However, these strategies target only a subset of many potential oncogenic proteins regulated by YAP1, which limit their overall effectiveness.^29^ Given the significant challenges in directly inhibiting YAP1, targeting upstream regulators of YAP1 activity or stability present more promising strategies for treating cancers. In this study, we reveal several novel insights with significant clinical implications for the treatment of CRC. First, we demonstrated that CDK4/6 inhibition by abemaciclib significantly destabilizes YAP1 and suppresses CRC progression. Next, DUB3 is identified as a novel DUB of YAP1, exhibiting its previously uncharacterized tumor‐promoting activity in CRC primarily through stabilizing YAP1. Furthermore, we discover that DUB3 phosphorylation and subsequent activation by CDK4/6 is a key mechanism to elevate YAP1 expressions in CRC. These findings indicate that CDK4/6 inhibition may represent a promising therapeutic strategy for the clinical management of CRC with upregulated DUB3 and YAP1 (Figure 6D).

Our FDA‐approved drug screening reveals that abemaciclib, a selective CDK4/6 inhibitor, effectively promotes YAP1 ubiquitination and degradation, thereby inhibiting CRC progression in CDX and PDX models (Figures 1 and S1). In response to proliferative signaling, CDK4/6 are activated, leading to the hyperphosphorylation of RB, which in turn activates E2F and facilitates cell cycle entry into S phase.^45^, ^46^ CDK4/6 inhibition demonstrates strong tumor‐suppressive effects with tolerable toxicity by inhibiting RB phosphorylation, preventing E2F release, and blocking the progression from G1 to S phase.^47^ Currently, three orally administered CDK4/6 inhibitors abemaciclib, palbociclib and ribociclib have received FDA approval for the treatment of HR^+^/HER2^−^ breast cancer patients.^46^ Beyond breast cancer, CDK4/6 inhibition also suppresses non–small‐cell lung cancer and melanoma.^48^, ^49^ Although the efficacy of CDK4/6 inhibition HR^+^/HER2^−^ breast cancer cells was well‐established, effects of targeting CDK4/6 in CRC remain largely unexplored. Interestingly, aberrant upregulation of CDK4 and cyclin D is commonly observed in CRC with enhanced dysplasia, correlating with increased tumor cell proliferation.^50^, ^51^ Thus, targeting CDK4/6 may be promising for treating CRC. Here, we revealed that CDK4/6 depletion or inhibition by abemaciclib significantly inhibited CRC cell proliferation, cell cycle progression, CSC self‐renewal, and chemoresistance both in vitro and in vivo in a YAP1‐dependent manner (Figures 1 and S1). However, it is noteworthy that G1 Therapeutics ended its CRC study of trilaciclib, another CDK4/6 inhibitor, due to lower survival outcomes. Trilaciclib has been FDA‐approved to decrease the incidence of bone marrow suppression in adults undergoing specific chemotherapy regimens for extensive‐stage small cell lung cancer.^52^ Despite all being CDK4/6 inhibitors, differences in chemical structures, transport and metabolism may lead to nonuniform antitumor effect and variable toxicities.^23^ For instance, the half‐life of trilaciclib is markedly shorter than those of palbociclib, ribociclib and abemaciclib.^23^, ^53^ Further preclinical studies and clinical studies are needed to explore the potentials of abemaciclib in managing CRC, particularly given that abemaciclib reduces YAP1 protein levels more significantly than trilaciclib and palbociclib (Figure 1B).

CDK4/6 has been shown to phosphorylate and affect the turnover of its substrates.^24^ Interestingly, CDK4/6's role in stabilizing YAP1 in CRC does not involve direct action on YAP1, since the interaction of purified GST‐CDK4/6 and His‐YAP1 was not detected (Figure 2A). While several DUBs, such as USP10 and USP9X, have been reported to affect YAP1 ubiquitination in HCCs and breast cancers respectively,^11^, ^21^ the deubiquitinating enzyme responsible for YAP1 regulation in CRC has remained unidentified. Here, we identified DUB3 as the novel DUB of YAP1 in CRC. DUB3 is known to control phosphorylated variant of histone 2AX (γH_2_AX) ubiquitination and regulate DNA damage response.^54^ Additionally, DUB3 stabilizes cyclin A and cdc25A, thereby regulating cell cycle progression.^55^, ^56^ Notably, we demonstrated that the inhibitory effects of abemaciclib on YAP1 stability and YAP1‐driven tumor progression are largely mediated by DUB3, a previously unidentified deubiqutinase of YAP1 in CRC. We found that the enzymatic activity of DUB3 is essential to directly deubiquitinate and stabilize YAP1, thus promoting YAP1‐mediated malignancies in vitro and in vivo (Figures 2, 3, and S2, 3). Mechanistically, CDK4/6 directly phosphorylates DUB3 at Ser41, activating DUB3 to deubiquitinate and stabilize YAP1 (Figure 4). Consistently, blocking Ser41 phosphorylation, either by CDK4/6 inhibition or the reconstitution of the Ser41A mutant, promotes YAP1 degradation and suppresses YAP1‐driven tumor progression (Figures 5 and S4). Clinically, our histological analysis shows a positive correlation between YAP1 and DUB3 expression in CRC specimens (Figure 6).

Our previous study showed that DUB3 deubiquitinates the key epithelial‐mesenchymal transition (EMT) factor Snail1, promoting tumor cell migration, invasion and metastasis of TNBC.^43^ However, Snail1 expression is only slightly higher in CRC tumor specimens than paired normal tissues (data not shown), suggesting that DUB3 might promote CRC progression primarily by stabilizing YAP1 rather than Snail1. Moreover, phosphorylation of DUB3 at Ser41 by CDK4/6 of CRC is crucial for its enzymatic activation in CRC, as the S41A mutant impairs DUB3's catalytic activity toward YAP1, regardless of CDK4/6 activity (Figures 5 and S4). Collectively, our findings underscore the tumor‐promoting function of the CDK4/6‐DUB3 axis in CRC through stabilizing YAP, suggesting that CDK4/6 inhibitors could be promising for CRC associated with aberrantly elevated DUB3 and YAP1.

Our study has some limitations. Firstly, Ser41 is located with in an unstructured region of DUB3. How CDK4/6‐catalyzed phosphorylation activates the deubiquitinase activity of DUB3 toward YAP1 in CRC remains elusive. Structural studies might be expected to provide more detailed evidence to support our proposed model herein by which the Ser41 phosphorylation of regulates DUB3's activity on YAP1 or some other substrates. Secondly, we cannot exclude the possibility of other mechanisms involved in DUB3‐mediated CRC progression, as the ectopic expression of YAP1 in CRC cells was unable to fully restore the functional changes induced by DUB3 depletion (Figures 3 and S3). It will be important to explore the potential involvements of other substrates of DUB3 other than YAP1 in CRC in future studies. Thirdly, although our preclinical evidence demonstrated the therapeutic potential of CDK4/6 inhibition CRC CDX and PDX xenograft models, clinical studies are essential to confirm the potentials of abemaciclib and other CDK4/6 inhibitors in CRC. Finally, besides CRC, upregulated YAP1, cyclin D, CDK4/6, and DUB3 are similarly implicated in various cancers such as lung cancer and ovarian cancers,^57^, ^58^, ^59^ thus the implication of this axis in these malignancies should be investigated by further preclinical and clinical study.

## 4 MATERIALS AND METHODS

### 4.1 Cell culture, plasmids, and antibodies

HEK293T, LoVo, HCT 116 cells were obtained from American Type Culture Collection (ATCC) and cultured in Dulbecco's modified Eagles's medium (Gibco), F‐12K Medium (Gibco) or in McCoy's 5a Medium Modified (Gibco) supplemented with 10% fetal bovine serum (FBS; Gibco). All cell lines used this study have been authenticated through Short Tandem Repeat (STR) analysis. Additionally, mycoplasma testing was performed using Mycoplasma PCR Detection Kit (C0301S, Beyotime) to ensure the absence of mycoplasma contamination.

CDK4, CDK6, DUB3, YAP1, CTGF, and CYR61 were cloned into pIRES (containing FLAG and S tag), pLV.3 (containing FLAG tag), pLV.5 (containing HA and S tag), pCMV‐HA (containing HA tag), PET28A (containing His tag), and pGEX4T‐1 (containing GST tag) vectors, respectively. All site mutants were generated through site‐directed mutagenesis and verified by sequencing.

In all experiments using shRNAs, pLKO.1‐scramble shRNA served as the negative control (CCTAAGGTTAAGTCGCCCTCG).

shRNA targeting sequences for

shCDK4: 5′‐GAGATTACTTTGCTGCCTTAA‐3′

shCDK6: 5′‐CAGATGTTGATCAACTAGGAA‐3′

shDUB3#1: 5′‐CACAAGCAGGtAGATCATCAC‐3′

shDUB3#2: 5′‐ GCAGGAAGATGCCCATGAATT‐3′

shYAP1#1: 5′‐GCAGACAGATTCCTTTGTTAA‐3′

shYAP1#2: 5′‐ CCACCAAGCTAGATAAAGAAA‐3′.

Antibodies against YAP1 (66900‐1‐Ig, 1:1000), DUB3 (26143‐1‐AP, 1:300), CYR61 (67656‐1‐Ig, 1:1000), CTGF (625474‐1‐AP, dilution: 1:1000) were from Proteintech Group. Phospho‐CDK substrate antibody (9477, 1:500), K48 or K63‐linkage Specific Polyubiquitin Rabbit mAb (8081, 5621, 1:1000) were from Cell Signaling Technology. Anticleaved‐PARP1 (CY5035, 1:1000) was purchased from ABways. Anti‐FLAG (m2, 1:1000), anti‐HA (H3663, 1:1000), and anti‐β‐actin (A1978, 1:5000) antibodies were from Sigma‐Aldrich. CDK4 (sc‐23896, 1:500), CDK6 (sc‐7961, 1:500), and Anti‐Ub (sc‐8017, 1:1000) antibodies were purchased from Santa Cruz Biotechnology, Inc. Anti‐pSer41 (1:100) was generated by immunizing rabbits with a phospho‐peptide, and subsequently affinity‐purified as described previously.^43^ For co‐IP experiments, heavy or light chain‐specific IPKine™ HRP (A25222 and A25022) were from Abbkine Scientific Co.

### 4.2 Screening of small molecular compounds analysis

Endogenous YAP1‐deficient HCT 116 cells were infected with lentiviruses encoding shRNA resistant GFP‐tagged YAP1 and treated with compound vehicle or compounds from the FDA‐approved drug library (1600 compounds) at 10 µM. At 24 h after treatment, the intensity of green fluorescence was determined. The levels of GFP‐YAP1 following treatment compound were quantified relative to vehicle.

### 4.3 Tandem affinity purification and mass spectrometry analyses

HCT116 cells stably expressing either empty vector or FLAG‐S‐tagged YAP1 were generated and subsequently treated with MG132 (10 µM) for 10 h. The cell pellets were lysed in NETN buffer (20 mM Tris–HCl, 300 mM NaCl, 1 mM ethylenediaminetetraacetic acid, 0.5% NP‐40, pH 8.0) supplemented with 1 × protease inhibitor cocktail (Roche), 1 mM sodium orthovanadate, 10 mM β‐glycerophosphate, 1 mM phenylmethylsulfonyl fluoride, and 10 mM sodium fluoride. Twenty microliter of Anti‐FLAG Affinity Gel (Sigma‐Aldrich) was added and rotated at 4°C for 2 h. Anti‐FLAG immunoprecipitates were washed three times with cold NETN buffer and incubated with 100 µL 3 × FLAG peptide working solution at the concentration of 100 ug/mL (Sigma‐Aldrich, F4799) at 4°C for 2 h. The elution process was repeated for three additional times, and the combined elutes were diluted by NETN buffer. S‐protein agarose (50 µL, Merck Millipore) was then added into the elutes and incubated at 4°C for 4 h. The immunoprecipitates bound to the S‐protein agarose were washed three times with cold NETN buffer. The beads were resuspended in 500 µL of 6 M urea in phosphate‐buffered saline (PBS), and 25 µL of 200 mM dithiothreitol (DTT) in 25 mM NH_4_HCO_3_ buffer was added. The reaction was then incubated at 37°C for 30 min. For alkylation, 25 µL of 400 mM Indole‐3‐acetic acid (IAA) in 25 mM NH_4_HCO_3_ buffer was added, and the mixture was incubated at room temperature in the dark for 30 min. For digestion, 150 µL of 2 M urea in PBS, 150 µL of 1 mM CaCl_2_ in 50 mM NH_4_HCO_3_, and 1 µL of trypsin (1.0 µg/µL) were added to the reaction, which was then incubated at 37°C overnight. The resulting peptides were collected by washing with 200 µL of water for three times and subsequently desalted using a C18 column. The evaporated samples were analyzed by liquid chromatography with tandem mass spectrometry (LC–MS/MS), utilizing an EASY‐nLC 1200 HPLC system coupled with ORBitrap Fusion Lumos mass spectrometer (Thermo Fisher Scientific). Raw data were processed using MaxQuant software (1.5.8.3), and searches were conducted against the UniProtKB human database (taxonomy 9606, version 20180929). Reversed database searches were performed to evaluate false discovery rate (FDR) of site, peptide, and protein identifications.

### 4.4 Denaturing immunoprecipitation for ubiquitination

Cell pellets were resuspended and lysed in 100 µL of lysis buffer 62.5 mM Tris–HCl (pH 6.8), 10% glycerol, 2% sodium dodecyl sulfate (SDS), 1 mM iodoacetamide, and 20 mM N‐ethylmaleimide. The lystaes were then boiled for 15 min, diluted 10 times with NETN buffer containing 1 × protease inhibitor cocktail (Roche), 20 mM N‐ethylmaleimide (NEM), and 1 mM iodoacetamide. After dilution, samples were centrifuged and subjected to immunoprecipitation using indicated antibodies. The resulting immunoprecipitates were then analyzed by western blotting.

### 4.5 in vitro ubiquitination assay

Indicated cells were transfected with empty vector or FLAG‐YAP1 followed with the treatment of MG132 (10 µM) for 10 h. YAP1 was immunoprecipitated using anti‐FLAG affinity gel.

*Escherichia coli* strain BL21 and Pierce Glutathione Agarose were used to express and purify recombinant GST‐fused DUB3 WT and C89S mutant protein. Ubiquitinated YAP1 protein isolated from indicated cells was then incubated separately with purified GST‐DUB3 WT or C89S fusion protein for 4 h. Western blotting was performed to analyze the ubiquitination of YAP1.

### 4.6 Quantitative real‐time PCR (qRT‐PCR)

RNA extraction from cultured cells was performed using TRIzol reagent (Thermo Scientific), and then RNA was subsequently reverse transcribed to cDNA using a FastKing gDNA Dispelling RT SuperMix (Tiangen). FastFire qPCR PreMix (SYBR Green) were used to perform qRT‐PCR analysis. All qRT‐PCR experiments were conducted in triplicate using GAPDH as an internal control. The primer sequences are as follows:

*YAP1* Forward 5′‐TGGGAGATGGCCAAGACATC‐3′, Reverse 5′‐CATGTTGTTGTCTGATCGTTGTGA‐3′.

*CTGF* Forward 5′‐AACCGCAAGATCGGAGTG‐3′, Reverse 5′‐ TGCTTTGGAAGGACTCACC‐3′.

*CYR61* Forward 5′‐TGAGTTAATCGCAATTGGAA‐3′, Reverse 5′‐ GTGGTCTGAACGATGCATTTC‐3′.

*ANKRD1* Forward 5′‐CACTTCTAGCCCACCCTGTGA‐3′, Reverse 5′‐CCACAGGTTCCGTAATGATTT‐3′.

*GAPDH* Forward 5′‐TGCACCACCAACTGCTTAG‐3′, Reverse 5′‐ CTTCTGGGTGGCAGTGATG‐3′.

### 4.7 Tumor sphere formation assay

The HCT 116 cells were suspended as single cells in stem‐cell culture medium consisting of FBS‐free DMEM/F12, supplemented with basic fibroblast growth factor (bFGF, 10 ng/mL), recombinant human epidermal growth factor (EGF) (rhEGF, 10 ng/mL), and N‐2 supplement.^60^

Five thousand cells were seeded into a low adhesion 24‐well plate containing 500 µL of stem‐cell culture medium, with three wells per group. After 2 weeks of culture, tumor spheres formed were counted, and their diameters were measured.

### 4.8 Animal studies

All animal experiments were conducted following a protocol approved by the Institutional Animal Care and Use Committee at JINAN University. For the subcutaneous xenograft experiment, HCT 116 cells (2 × 10^6^) were subcutaneously implanted into female BALB/c nude mice (5–6 weeks old, *n* = 6; Jicui Yaokang Biotechnology Co., Ltd.). Tumor volumes were measured three times per week using a vernier caliper. The volumes were calculated using the formula: width^2^ × length × 0.4 (mm^3^). When tumor sizes reached 150–200 mm^3^, mice were randomized and administered one of the following treatments: saline, abemaciclib (50 mg/kg/day), or cisplatin (2 mg/kg, once a week). Treatments were continued for the predetermined duration. PDXs were established following the protocol approved by IACUC at JINAN University (20230205‐19) according to the previous studies.^61^ Briefly, CRC PDXs were cut into pieces of 3 × 3 × 3 mm^3^ using sterile surgical instruments and tumor blocks were insert into the back of mice by inoculation needle. Tumor volumes were measured three times weekly. For lentivirus injection, lentiviruses were produced in HEK293T cells with indicated transfection. Then lentiviruses were filtered through a 0.45‐µm filter and concentrated using PEG 8000 precipitation. When tumor volumes reached 30–50 mm^3^, the center of the xenograft tumors were intratumorally injected with lentivirus (1 × 10^8^ PFU/100 µL per mouse) for three times. When tumor volumes reached 150–200 mm^3^, saline, abemaciclib (50 mg/kg/day), or cisplatin (2 mg/kg, once a week) was administered (*n* = 6). Mice were euthanized at indicated time and tumor weights were measured.

### 4.9 Immunohistochemical staining

A total of 58 sets of CRC tissues along with paired normal colorectal tissues were obtained from the tissue bank at the First Affiliated Hospital of Jinan University, following approval from the Institutional Medical Ethics Committee (Ethics Approval License: JNUKY‐2022‐098). Seventeen pairs of samples were utilized for western blotting assay and 58 sets of samples were used for the IHC staining. IHC assays were conducted on paraffin‐embedded specimens of CRC patients using anti‐YAP1, anti‐DUB3 antibodies, respectively. The immunohistochemical staining and quantification were performed as previously described.^62^ The immunostaining results were blindly evaluated by two independent pathologists, and IHC scores were calculated according to the method outlined in a previous study.^63^ For statistical analysis, the chi‐square test (*χ* ^2^‐test) and the Pearson's correlation coefficient were used to assess the correlation between YAP1 and DUB3 expression. The relationship of clinical data was analyzed using the *χ* ^2^‐test by SPSS 22.0.

### 4.10 Statistical analysis

All in vitro experiments were conducted independently three times to ensure repeatability. In the animal studies, data are expressed as the mean ± SD from six mice. GraphPad Prism software version 9.3 was used for statistical analyses. One‐way analysis of variance (ANOVA) analysis followed by Tukey's test or *t*‐test was utilized to compare results with significance levels defined as ^ns^ *p* > 0.05; ^*^p < 0.05; ^**^p < 0.01; ^***^p < 0.001. The relationship of clinical data was assessed using the *χ* ^2^‐test.

## AUTHOR CONTRIBUTIONS

Tongzheng Liu conceived the study and designed the experiments. Yalei Wen performed most experiments with assistance from Xiao Yang, Lei Huang, Jiayi Chen, Lirong Tan, Xiuqing Ma, Yingjie Zhu. Shengrong Li and Zhengqiu Li conducted the LC‐MS/MS‐based experiments and analyzed data. Tongzheng Liu, Yalei Wen, and Xiao Yang wrote the manuscript with input from Qiushi Zhang, Mingchao Liang, and Haoxing Zhang. Changliang Shan and Chunze Zhang provided the CRC PDX. Tongzheng Liu guided and supervised the study. All the authors read and approved the final manuscript.

## CONFLICT OF INTEREST STATEMENT

The authors declare no conflicts of interest.

## ETHICS STATEMENT

CRC pathological tissue sections were obtained from the tissue bank at The First Affiliated Hospital of Jinan University in accordance with the approval document of the Institutional Medical Ethics Committee (JNUKY‐2022‐098). All animal experiments were performed in accordance with a protocol approved by the Institutional Animal Care and Use Committee of the JINAN University (20230205‐19). Written informed consent was obtained from all participants.

## Supporting information



Supporting Information

## Data Availability

All data associated with this study are present in the paper or the Supporting Information. Information about all commercially available reagents is provided in Section 4.
