# Supplementary material for: Targeting CDK4/6 suppresses colorectal cancer by destabilizing YAP1
Source: MedComm (2020). 2025 Feb 17;6(3):e70103. doi: 10.1002/mco2.70103 (PMC11832431; doi:10.1002/mco2.70103)
Supplement: Supplementary file 1 — Supporting Information [file MCO2-6-e70103-s001.docx]

# Targeting CDK4/6 Suppresses Colorectal Cancer by Destabilizing YAP1

# Short running title: CDK4/6 Inhibition Destablizes YAP1

**Supplementary Methods**

**Western blotting**

Cells were lysed in NETN buffer (pH 8.0, 300 mM NaCl, 20 mM Tris-HCl, 0.5% NP-40, 1 mM ethylenediaminetetraacetic acid (EDTA)) containing protease inhibitors (1× protease inhibitor cocktail (Roche), 1 mM sodium orthovanadate, 10 mM β-glycerophosphate, 1 mM phenylmetnylsulfonyl fluoride, and 10 mM sodium fluoride). Proteins were separated by SDS-PAGE gel electrophoresis and transferred to PVDF membranes, then incubated with the indicated primary and secondary antibodies.

**Immunoprecipitation**

Cells transfected with indicated plasmids were lysed in NETN buffer and were incubated 2 h with anti-FLAG affinity gel for 4 h at 4 °C. HCT 116 or LoVo cells as indicated were lysed in NETN buffer and were incubated overnight with primary antibodies together with protein A/G beads at 4 ℃. The immunoprecipitates were subjected to western blotting after washing beads for three times.

**Glutathione-S-transferase (GST) pull-down Assay**

Recombinant GST-CDK4/6 or GST-DUB3 and His-DUB3 or His-YAP1 proteins were expressed in Escherichia coli strain BL21. GST, GST-CDK4/6 or GST-DUB3 protein was purified using Pierce Glutathione agarose. Fusion proteins were mixed for 4 h at 4 °C as indicated. Beads were washed four times, and proteins were detected by western blotting.

**Cell proliferation assay**

LoVo and HCT 116 cells (4 ×10^4^) were seeded in each well. Cells from each well were digested with 0.25% trypsin at 37 ℃ the next day. And cell pellets were collected by centrifugation, washed by PBS, re-suspended in PBS and counted under microscope. Likewise, cell numbers were counted for the next 8 days with similar method.

**CCK-8 Assay**

A Cell Counting Kit-8 (HY-K0301) was used to measure the survival of LoVo and HCT 116 cells. A total of 2000 cells in a 100 μL medium containing 10% FBS were cultured in three replicate wells in a 96-well plate. Cells were then treated with different concentrations of cisplatin or 5-FU for 72 h and CCK-8 reagent (15 μL) was added and incubated for 2 h. The optical density values at a wavelength of 450 nm were measured using a microplate reader to determine cell viability.

**Flow Cytometry Analysis**

Cells stably expressing control or shDUB3#1, shDUB3#2 were treated with vehicle or abemaciclib (5 μM) and digested with 0.25% trypsin at 37 °C. Cell pellets were collected by centrifugation and then washed with PBS for two times. Cells were stained with antibodies anti‐CD44‐FITC (FHF0442-025, 4A Biotech, dilution: 1:100) and anti‐CD133-PE/Cyanine7 antibody (372810, BioLegend, dilution: 1:100) at 4 °C in the dark for 1 h. The pellets were then washed with PBS twice and re‐suspended in 500 µL PBS. Flow cytometry analysis was performed with a FACS LSRFortessa flow cytometer.

Cell Cycle Analysis Kit (no. C1052; Beyotime, Shanghai, China) was used in cell cycle assay. Cells were harvested and fixed in 70% ethanol for 2 h at 4 °C and then stained with a solution containing propidium iodide (0.05 mg/ml), RNase A (1 mg/ml), and 0.3% Triton X-100 in the dark for 30 min. The percentage of cells in different phases of the cell cycle was examined by measuring the DNA content (propidium iodide intensity) with a FACS LSRFortessa flow cytometer, and populations of G1, S, and G2/M phase cells were determined with the ModFIT software. Each experiment was repeated three independent times.

**Supplementary Figures**


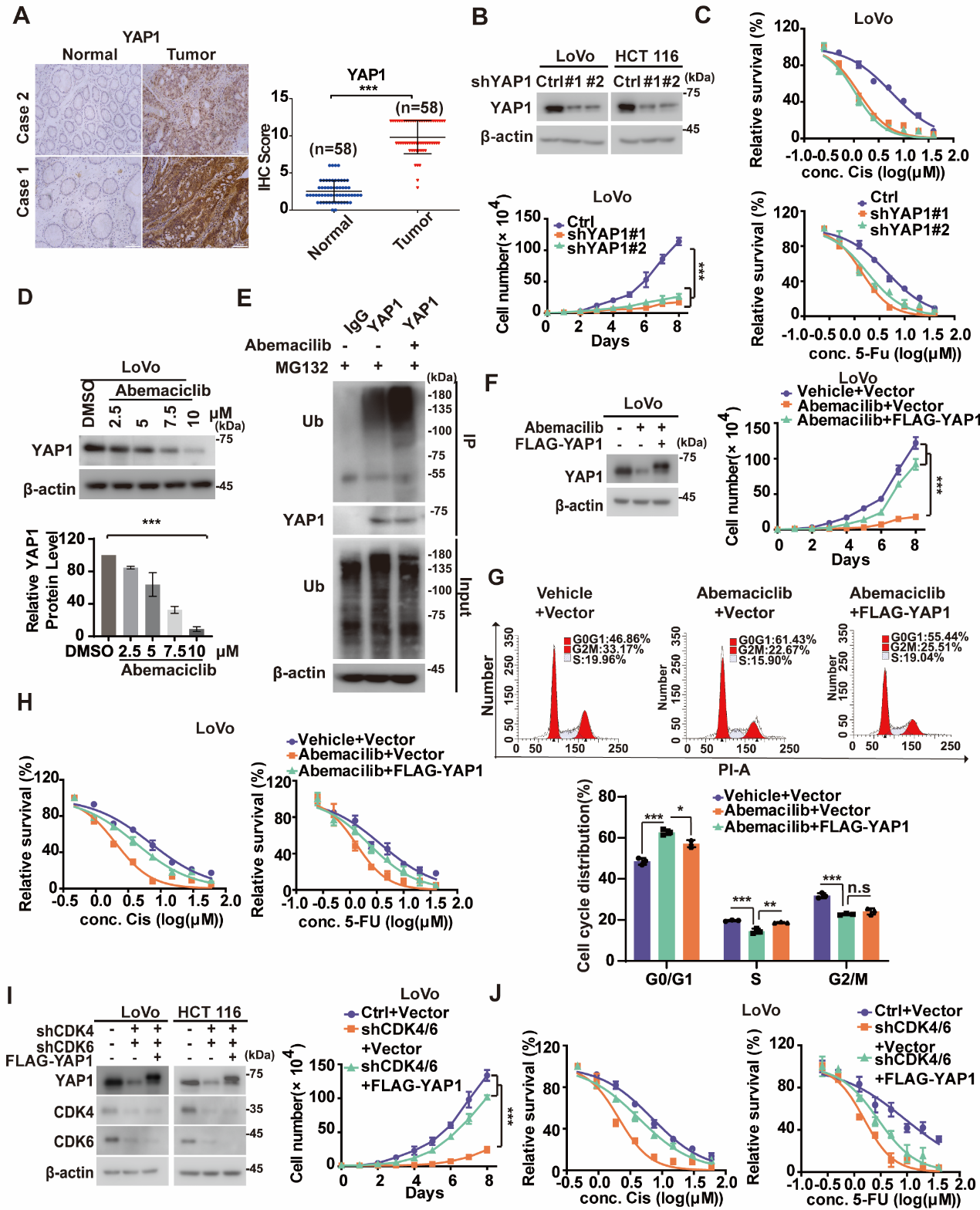

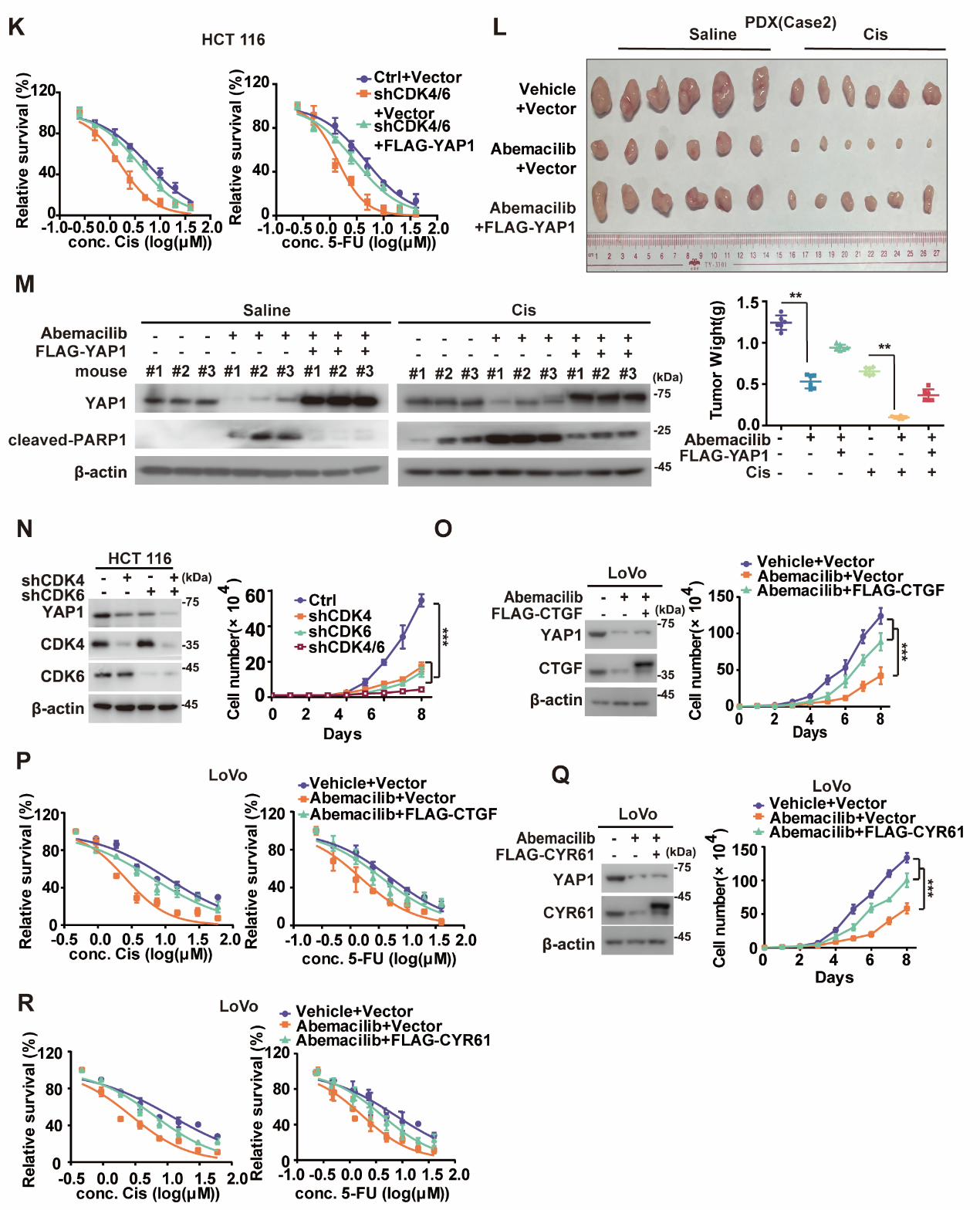


**Figure S1** CDK4/6 inhibition induces YAP1 degradation and suppresses colorectal cancer progression

A) Representative staining of YAP1 in 58 paired colorectal cancer and adjacent normal tissues. The analysis of YAP1 IHC score was showed in right panel. B) LoVo and HCT 116 cells stably expressing control (Ctrl), shYAP1#1, shYAP1#2 were generated and theprotein level of YAP1 was measured by western blotting. Cell proliferation assay was performed. Results represent the mean ± s.d. of three independent experiments. C) LoVo Cells as in (B) were treated with indicated concentrations of cisplatin or 5-FU and cell survival was determined. The results represent mean ± s.d. from three independent experiments. D) LoVo cells were treated with DMSO and different concentrations of Abemaciclib for 24 h and western blotting was performed with indicated antibodies. The relative level of YAP1 to β-actin was measured by image J. Results represent the mean ± s.d. of three independent experiments. E) LoVo cells were treated with DMSO or Abemaciclib (5 µM) for 24 h and then treated with MG132 (10 µM) for additional 10 h and cell lysates were subjected to immunoprecipitation with IgG or anti‐YAP1. The ubiquitination level of YAP1 was detected by western blotting. F) LoVo cells were transfected with indicated plasmids and treated with vehicle or abemaciclib (5 μM). Western blotting was performed with indicated antibodies. Cell proliferation assay was performed. The results represent mean ± s.d. from three independent experiments. G) Flow cytometry analysis showing the cell-cycle distribution of LoVo cells as in (F). The results represent mean ± s.d. from three independent experiments. H) Cells as in (F) were treated with indicated concentrations of cisplatin or 5-FU and cell survival was determined. The results represent mean ± s.d. from three independent experiments. I) LoVo and HCT 116 cells stably expressing control (Ctrl), shCDK4, shCDK6 were transfected with indicated plasmids. Western blotting was performed with indicated antibodies. Cell proliferation of cells were examined. Results represent the mean ± s.d. of three independent experiments. J-K) Cells as in (I) were treated with indicated concentrations of cisplatin or 5-FU and cell survival was determined. The results represent mean ± s.d. from three independent experiments. L) Colorectal cancer patient-derived xenografts (PDXs) were subcutaneously implanted into nude mice (5-6 weeks, n=6). When tumors reached around 150-200 mm3 in size, mice were treated with saline, abemaciclib (50 mg/kg), cisplatin (2 mg/kg) or abemaciclib plus cisplatin (n=6). Tumors were collected and tumor weights were analyzed. M) The PDX tumors as in (L) were homogenized and western blotting was performed with indicated antibodies. N) HCT 116 cells stably expressing control (Ctrl), shCDK4, shCDK6 or shCDK4/6 were transfected with indicated plasmids. Western blotting was performed with indicated antibodies. Cell proliferation of cells were examined. Results represent the mean ± s.d. of three independent experiments. O) LoVo cells were transfected with indicated plasmids and treated with vehicle or abemaciclib (5 μM). Western blotting was performed with indicated antibodies. Cell proliferation assay was performed. Results represent the mean ± s.d. of three independent experiments. P) Cells as in (O) were treated with indicated concentrations of cisplatin or 5-FU and cell survival was determined. The results represent mean ± s.d. from three independent experiments. Q) LoVo cells were transfected with indicated plasmids and treated with vehicle or abemaciclib (5 μM). Western blotting was performed with indicated antibodies. Cell proliferation assay was performed. Results represent the mean ± s.d. of three independent experiments. R) Cells as in (Q) were treated with indicated concentrations of cisplatin or 5-FU and cell survival was determined. The results represent mean ± s.d. from three independent experiments. *^n.s,^ P* ＞ 0.05; *^*,^ P* < 0.05; *^**,^ P* < 0.01; *^***,^ P* < 0.001 by Student's *t*-test or one-way ANOVA with Tukey's post hoc test.


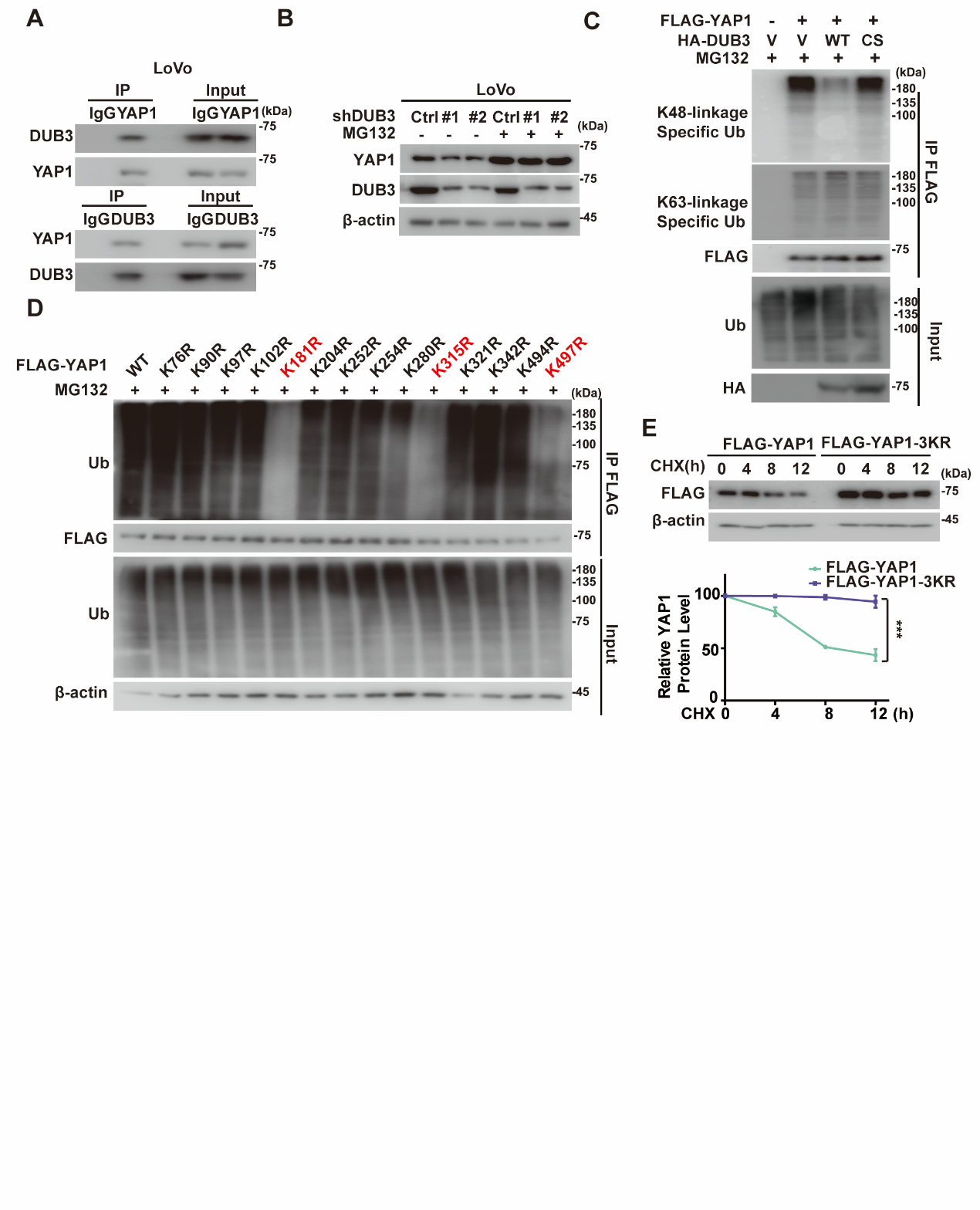


**Figure S2** DUB3 deubiquitinates and stabilizes YAP1

A) Cell lysates of LoVo were subjected to immunoprecipitation with IgG, anti-YAP1 or anti-DUB3 antibodies, respectively. Western blotting was performed with indicated antibodies. B) LoVo cells stably expressing control, shDUB3#1, shDUB3#2 were treated with vehicle or MG132 (10 μM) and western blotting was performed with indicated antibodies. C) Cells were transfected with indicated plasmids and treated MG132 (10 μM) for 10 h. YAP1 was immunoprecipitated with anti-FLAG affinity gel and the polyubiquitylated YAP1 protein was examined by K48- or K63-linkage specific polyubiquitin antibodies. D) Cells were transfeced with vector, pIRES-YAP1 WT or different KR mutants (containing FLAG and S tag) and then treated with MG132 (10 μM) for 10 h. YAP1 was immunoprecipitated with anti-FLAG affinity gel and the polyubiquitylated YAP1 protein was examined by western blotting. E) LoVo cells were transfected with pIRES-YAP1 or the 3KR (K181R/K315R/K497R) mutant (containing FLAG and S tag) and cycloheximide pulse-chase assay was performed. The relative level of FLAG-YAP1 to β-actin was measured by image J. Results represent the mean ± s.d. of three independent experiments. *^n.s,^ P* ＞ 0.05; *^*,^ P* < 0.05; *^**,^ P* < 0.01; *^***,^ P* < 0.001 by Student's *t*-test.


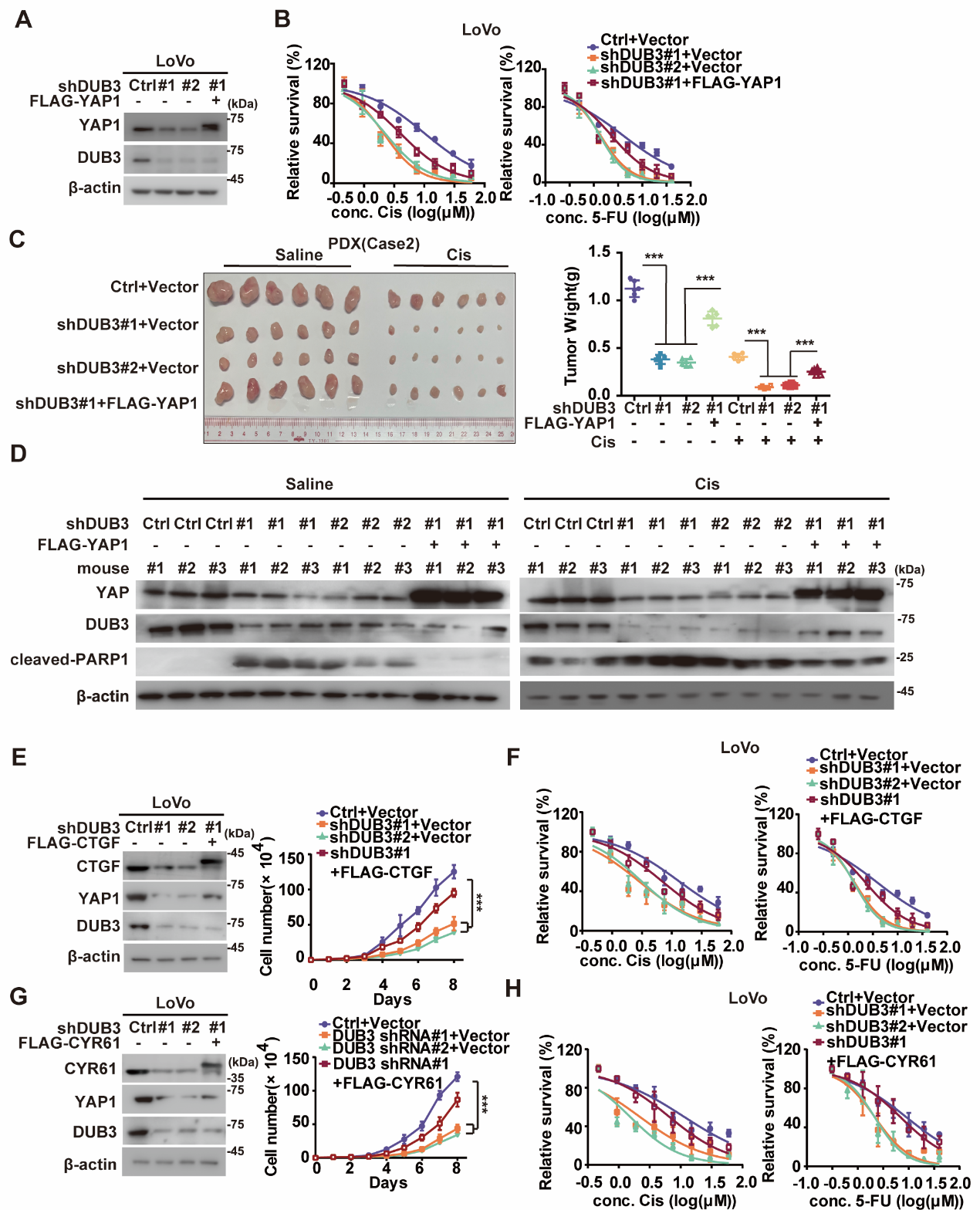


**Figure S3** DUB3 promotes CRC tumor progression through stabilizing YAP1

A) LoVo cells stably expressing Ctrl, shDUB3#1, shDUB3#2 were transfected with vector or FLAG-YAP1 and western blotting was performed with indicated antibodies. B) LoVo cells as in (A) were treated with indicated concentrations of cisplatin or 5-FU and cell survival was determined. Results represent mean ± s.d. from three independent experiments. C) Colorectal cancer patient-derived xenografts (PDX case 2) were subcutaneously implanted into nude mice (5-6 weeks, n=6). Xenograft tumors were injected with lentivirus expressing the indicated constructs when tumor volume reached 30-50 mm3. Mice were then treated with saline or cisplatin (2 mg/kg), respectively. Tumors were collected and tumor weights were analyzed. Xenograft tumors were dissected at indicated time and tumor weights were measured in right panel. Results represent the mean ± s.d. from six mice. D) The PDX tumors as in (C) were homogenized and western blotting was performed with indicated antibody. E) LoVo cells stably expressing Ctrl, shDUB3#1, shDUB3#2 were transfected with vector or FLAG-CTGF and western blotting was performed with indicated antibodies Cell proliferation assay was performed. Results represent the mean ± s.d. of three independent experiments. F) Cells as in (E) were treated with indicated concentrations of cisplatin or 5-FU and cell survival was determined. The results represent mean ± s.d. from three independent experiments. G) LoVo cells stably expressing Ctrl, shDUB3#1, shDUB3#2 were transfected with vector or FLAG-CTGF and western blotting was performed with indicated antibodies Cell proliferation assay was performed. Results represent the mean ± s.d. of three independent experiments. H) Cells as in (G) were treated with indicated concentrations of cisplatin or 5-FU and cell survival was determined. The results represent mean ± s.d. from three independent experiments. *^n.s,^ P* ＞ 0.05; *^*,^ P* < 0.05; *^**,^ P* < 0.01; *^***,^ P* < 0.001 b one-way ANOVA with Tukey's post hoc test.


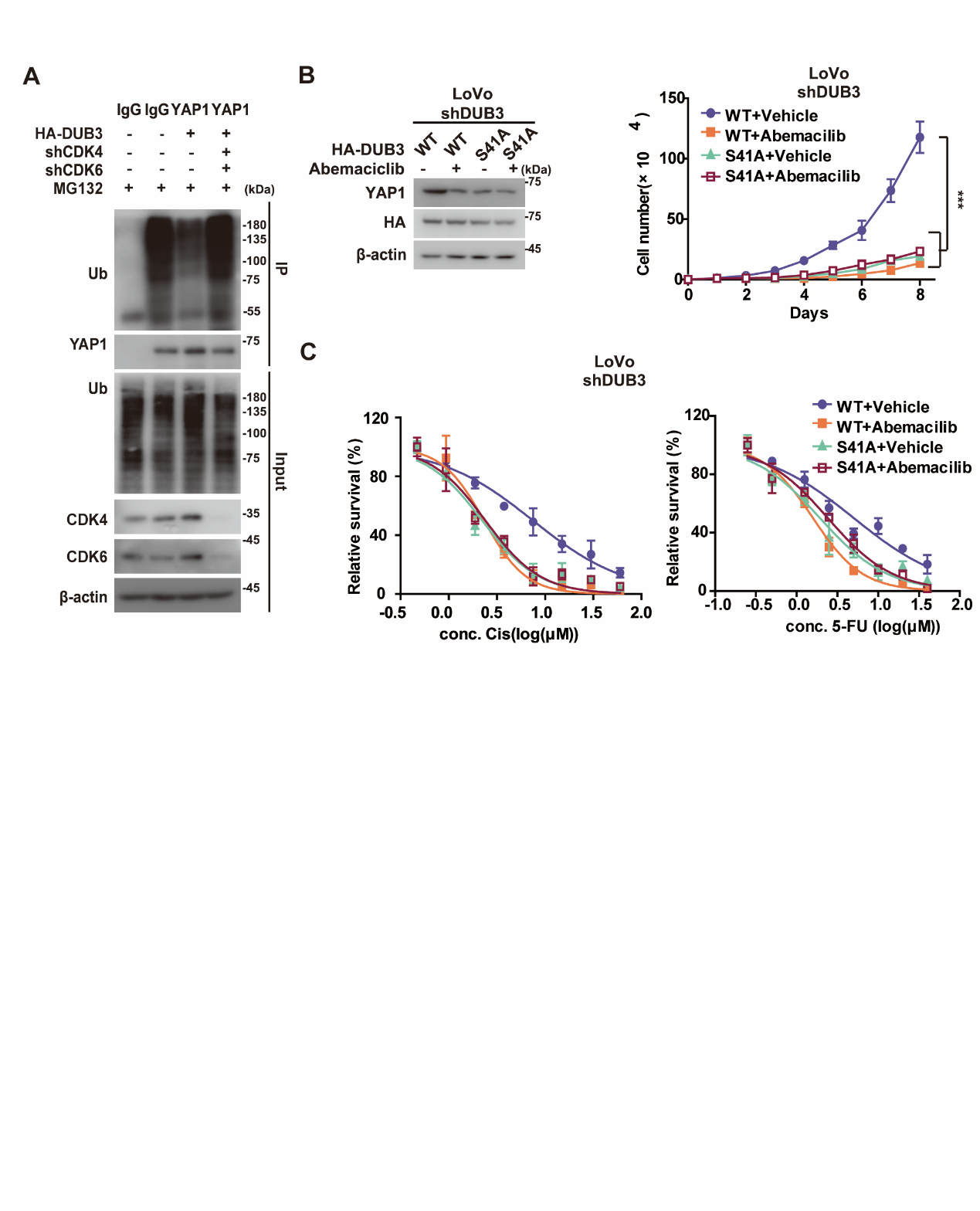


**Figure S4** CDK4/6-mediated phosphorylation of DUB3 regulates YAP1 degradation and CRC progression

A) Cells were transfected with indicated plasmids and then treated with MG132 (10 μM) for 10 h and cell lysates were subjected to immunoprecipitation with IgG or anti‐YAP1. The ubiquitination level of YAP1 was detected by western blotting. B) Endogenous-DUB3 deficient LoVo cells were transfected with indicated plasmids. The protein level of YAP1 was determined by western blot and cell proliferation was measured. The results represent mean ± s.d. from three independent experiments. C) LoVo cells as in (B) were treated with indicated concentrations of cisplatin or 5-FU and cell survival was determined. The results represent mean ± s.d. from three independent experiments. *^n.s,^ P* ＞ 0.05; *^*,^ P* < 0.05; *^**,^ P* < 0.01; *^***,^ P* < 0.001 b one-way ANOVA with Tukey's post hoc test.


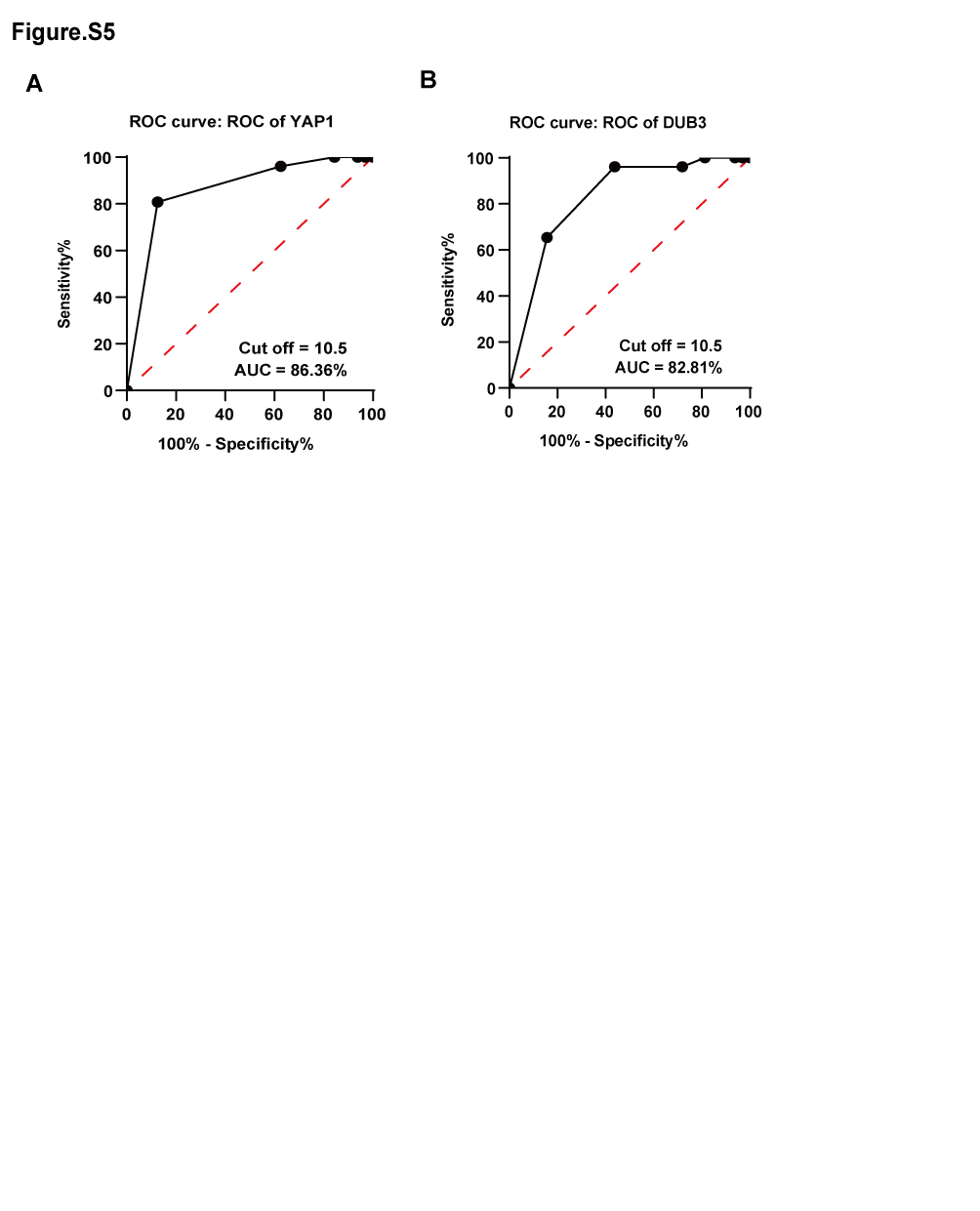


**Figure S5** Receiver operating characteristic (ROC) curve of DUB3 and YAP1

A-B) ROC curve analysis showed that the optimal cut-off score of YAP1(A) and DUB3(B) to predict clinical stage with high sensitivity and specificity was both 10.5 in CRC patients (n=58).
